# Diagnosis and treatment of hyponatremia: a systematic review of clinical practice guidelines and consensus statements

**DOI:** 10.1186/s12916-014-0231-1

**Published:** 2014-12-11

**Authors:** Evi V Nagler, Jill Vanmassenhove, Sabine N van der Veer, Ionut Nistor, Wim Van Biesen, Angela C Webster, Raymond Vanholder

**Affiliations:** European Renal Best Practice (ERBP), guidance body of the European Renal Association – European Dialysis and Transplant Association (ERA-EDTA), De Pintelaan 185, Ghent, 9000 Belgium; Renal Division, Department of Internal Medicine, Ghent University Hospital, De Pintelaan 185, Ghent, 9000 Belgium; Centre for Kidney Research, The Children’s Hospital at Westmead, Locked Bag 4001, Westmead 2145, NSW Australia; Department of Medical Informatics, Academic Medical Center, Postbus 22660, Amsterdam, 1100 DD the Netherlands; Nephrology Department, Gr. T. Popa University of Medicine and Pharmacy, Strada Universitãtii 16, Iasi, 700115 Romania; Sydney School of Public Health, Edward Ford Building (A27), The University of Sydney, NSW, 2006 Australia; Centre for Transplant and Renal Research, The University of Sydney at Westmead Hospital, Westmead 2145, NSW Australia

**Keywords:** Clinical practice guideline, Hyponatremia, Systematic review

## Abstract

**Background:**

Hyponatremia is a common electrolyte disorder. Multiple organizations have published guidance documents to assist clinicians in managing hyponatremia. We aimed to explore the scope, content, and consistency of these documents.

**Methods:**

We searched MEDLINE, EMBASE, and websites of guideline organizations and professional societies to September 2014 without language restriction for Clinical Practice Guidelines (defined as any document providing guidance informed by systematic literature review) and Consensus Statements (any other guidance document) developed specifically to guide differential diagnosis or treatment of hyponatremia. Four reviewers appraised guideline quality using the 23-item AGREE II instrument, which rates reporting of the guidance development process across six domains: scope and purpose, stakeholder involvement, rigor of development, clarity of presentation, applicability, and editorial independence. Total scores were calculated as standardized averages by domain.

**Results:**

We found ten guidance documents; five clinical practice guidelines and five consensus statements. Overall, quality was mixed: two clinical practice guidelines attained an average score of >50% for all of the domains, three rated the evidence in a systematic way and two graded strength of the recommendations. All five consensus statements received AGREE scores below 60% for each of the specific domains.

The guidance documents varied widely in scope. All dealt with therapy and seven included recommendations on diagnosis, using serum osmolality to confirm hypotonic hyponatremia, and volume status, urinary sodium concentration, and urinary osmolality for further classification of the hyponatremia. They differed, however, in classification thresholds, what additional tests to consider, and when to initiate diagnostic work-up. Eight guidance documents advocated hypertonic NaCl in severely symptomatic, acute onset (<48 h) hyponatremia. In chronic (>48 h) or asymptomatic cases, recommended treatments were NaCl 0.9%, fluid restriction, and cause-specific therapy for hypovolemic, euvolemic, and hypervolemic hyponatremia, respectively. Eight guidance documents recommended limits for speed of increase of sodium concentration, but these varied between 8 and 12 mmol/L per 24 h. Inconsistencies also existed in the recommended dose of NaCl, its initial infusion speed, and which second line interventions to consider.

**Conclusions:**

Current guidance documents on the assessment and treatment of hyponatremia vary in methodological rigor and recommendations are not always consistent.

**Electronic supplementary material:**

The online version of this article (doi:10.1186/s12916-014-0231-1) contains supplementary material, which is available to authorized users.

## Background

Hyponatremia is the most common electrolyte disorder in clinical medicine; it represents an excess of water relative to total body solute [1]. Hyponatremia usually results from the intake and subsequent retention of electrolyte-free water in response to true hypovolemia due to gastrointestinal solute loss or malnutrition; decreased effective circulating volume due to heart failure or liver cirrhosis; or non-osmotic vasopressin activity due to malignancies, infections, medications, pain, or stress [2]. When defined as a serum sodium concentration below 135 mmol/L, hyponatremia occurs in up to 8% of the general population and in up to 60% of hospitalized patients [2,3]. Acute profound hyponatremia can cause brain edema, but also chronic mild hyponatremia is associated with poor health outcomes. Even when comorbid conditions are taken into account, people with a mildly decreased serum sodium concentration have a 30% higher risk of death and are hospitalized 14% longer relative to those without hyponatremia [2,4].

Despite the frequency and severity of some of the associated complications, research suggests hyponatremia is often neglected by clinicians [5]. If acquired in hospital, it may take days before the electrolyte disorder is investigated, potentially allowing a further decrease in serum sodium concentration and exposing patients to the dangers of profound hyponatremia. When efforts are made to explore the underlying cause, clinicians use widely different strategies for differential diagnosis, testing is often inadequate and misclassification of the hyponatremia frequently occurs [6,7].

Hyponatremia may be managed clinically by different specialists, such as endocrinologists, nephrologists, geriatricians, or intensivists, and, accordingly, management strategies often vary [5]. Although probably related to variation in awareness, differences in expert opinion on whom and how to treat only add to the confusion over optimal management. For instance, although experts agree that acute symptomatic hyponatremia should be treated with hypertonic saline, the optimal concentrations and methods for determining initial infusion speeds are debated [1]. In addition, the risk of osmotic demyelination syndrome after rapid correction of hyponatremia has fuelled intense debate among experts on whether complications of untreated hyponatremia or complications of treatment pose the greatest risk [8]. As different specialist physicians deal with hyponatremia, consultation of different information and guidance sources may add to the variability in treatment seen in clinical practice today.

Clinical practice guidelines and consensus statements provide recommendations to help evidence-based practice by suggesting the most appropriate diagnostic tests and the most appropriate treatments. Over the years, multiple organizations have developed recommendations to assist clinicians in the management of hyponatremia. To be reliable, these recommendations must be based on a systematic review of the evidence, and have a transparent and multidisciplinary development process [9]. Inconsistencies between recommendations may arise from failing to meet development standards and can only add to unwarranted variability in management. In this study, we aimed to explore the scope, content, and consistency of the existing guidance documents on the diagnosis and management of hyponatremia in adults and children.

## Methods

### Criteria for selection of studies

We included evidence-based clinical practice guidelines and consensus statements on the diagnosis and treatment of hyponatremia. We defined clinical practice guidelines as statements that included recommendations intended to optimize patient care informed by a systematic review of evidence and an assessment of the benefits and harms of alternative care options [9]. We defined consensus statements as documents containing clinically relevant suggestions or recommendations based on the collective opinion of an expert panel [9]. We included all publications independent of language. We excluded guidelines related to the prevention of hyponatremia as well as guidelines relevant to conditions associated with hyponatremia if they were not specifically designed to address hyponatremia. Hence, we excluded guidelines targeting treatment of heart failure, cirrhosis, and cancer unless they were developed with a focus on hyponatremia as a complication. Finally, we also excluded draft unpublished guidelines, conference or discussion papers, personal opinions, and obsolete guidelines replaced by updated recommendations from the same organization.

### Search methods for guidelines and consensus statements

We searched MEDLINE (1946 to September Week 1, 2014) and EMBASE (1980 to September 2014), combining vocabulary terms and text words for hyponatremia with terms related to clinical practice guidelines and consensus statements. We also searched guideline databases and websites of organizations as well as of selected professional specialist societies in nephrology, endocrinology, and intensive care medicine. A list of the databases and websites along with the full search strategies are outlined in Additional file 1. EN and JV independently screened the titles and abstracts and discarded those that did not meet the inclusion criteria. Full texts for potentially relevant guidelines or consensus statements were retrieved and examined for eligibility. Both the initial screening and subsequent full-paper assessment stage were completed using Early Review Organizing Software [10].

### Data collection process and data items

We developed a draft data extraction form which was piloted and modified as necessary. The extracted data included document characteristics (e.g., year of publication, country/region, development team, funding organization), recommendations related to the diagnosis and assessment of hyponatremia, and recommendations related to the treatment of hyponatremia. EN and JV extracted all data using the standardized data extraction form (Additional file 2) and resolved discrepancies by consensus.

### Appraisal of guidelines and consensus statements

Four reviewers independently assessed methodological quality using the Appraisal of Guidelines for Research and Evaluation (AGREE II) instrument [11]. AGREE II is an internationally validated, rigorously developed 23-item tool used to evaluate six domains of guideline development: scope and purpose, stakeholder involvement, rigor of development, clarity of presentation, applicability, and editorial independence [12] (Additional file 3). The AGREE tool has also been used to assess consensus statements [13,14]. The reviewers rated each item on a Likert scale from 1 (‘Strongly Disagree’) to 7 (‘Strongly Agree’). We calculated a total score for each domain by summing up all the scores of the individual items in a domain for each reviewer and then standardizing this total as a percentage of the maximum possible score for that domain, calculated as follows [12]:

$$ \frac{Obtained\kern0.5em  score- Minimum\kern0.5em  possible\kern0.5em  score}{Maximum\kern0.5em  possible\kern0.5em  score- Minimum\kern0.5em  possible\kern0.5em  score}*100\% $$

The minimum possible score for each domain equaled the number of questions multiplied by the number of reviewers, multiplied by 1 (strongly disagree). The maximum score for a domain equaled the number of questions multiplied by the number of reviewers, multiplied by 7 (strongly agree). To ensure standardization of each reviewer’s approach, all reviewers completed the online training tutorial [15] before starting the project.

In a consensus meeting among the reviewers, we discussed every item for which scores differed by more than 1 point (e.g., 1 versus 3) on the original 7-point scale. Reviewers in turn explained the rationale for their score and had the opportunity to revise their score when they considered this appropriate. We audiotaped the consensus meeting to reliably record the underlying reasons for changing scores.

### Synthesis of guideline recommendations

We conducted a textual descriptive synthesis to analyze the scope, content, and consistency of the included recommendations. EN inductively coded the text manually to identify domains covered by the guidelines. These were cross-tabulated with the guidelines and recommendations were inserted into the corresponding cell. For each domain, we compared guideline recommendations to identify similarities and discrepancies. Consistent with the scope of this review, we only tabulated the information on diagnosis and treatment of hyponatremia.

## Results

### Search results

**Figure 1:**
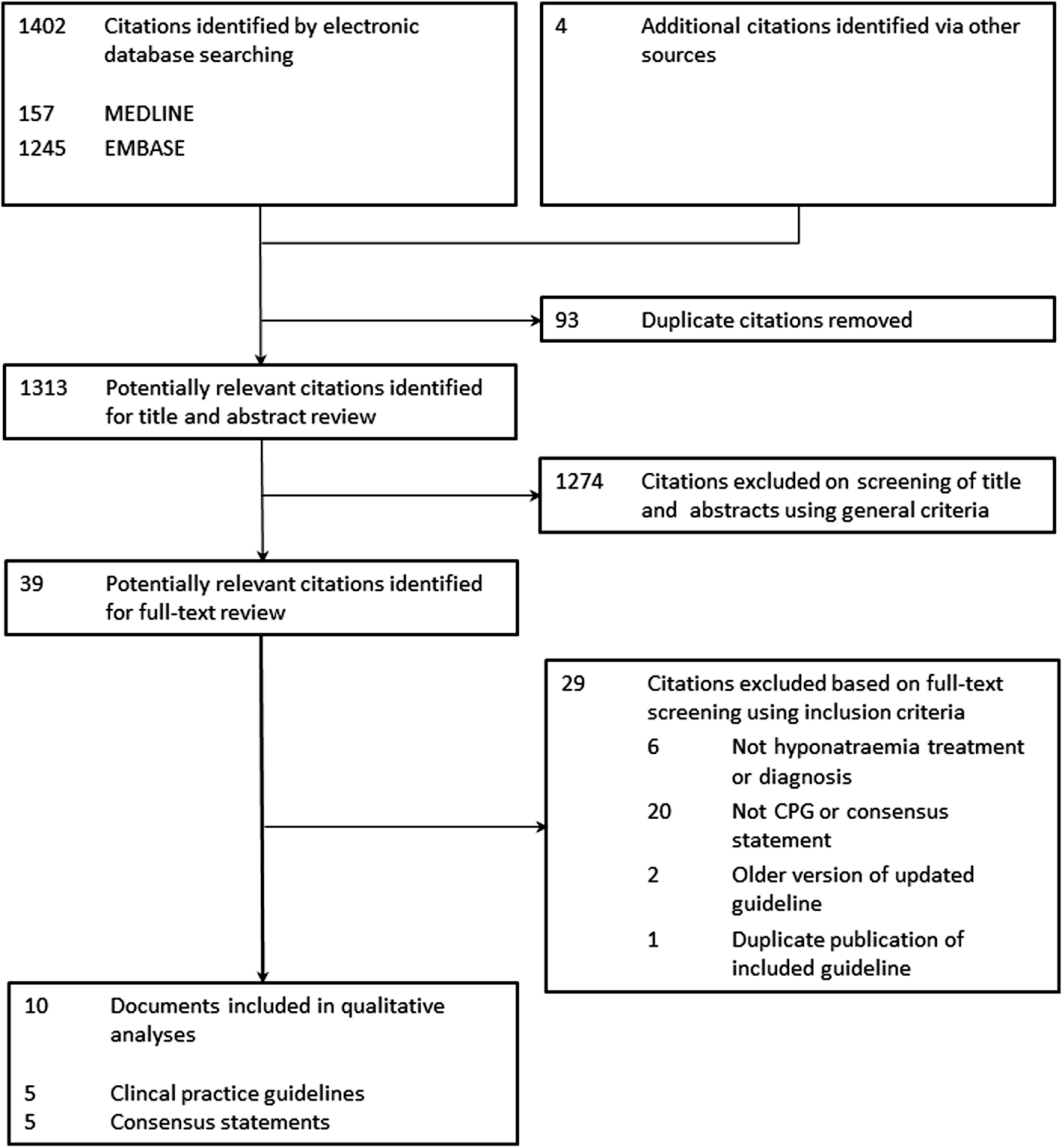
**Flow diagram of the identification process for clinical practice guidelines and consensus statements on hyponatremia.**

We identified 1,402 citations, of which we excluded 1,367 after screening titles and abstracts because they did not meet our eligibility criteria (Figure 1). We assessed the full text of the remaining 39 citations and excluded 29 because they were not related to the diagnosis or treatment of hyponatremia, were not clinical practice guidelines or consensus statements, or were guidelines replaced by an updated version (Additional file 4). Ultimately, we included five clinical practice guidelines [16-20] and five consensus statements [21-25]. Six of these documents were retrieved through searching the medical databases [18-20,23-25], the other four through the search of guideline databases and professional society websites [16,17,21,22].

Table 1 shows the general characteristics of the included clinical practice guidelines and consensus statements. Eight national or regional organizations from the Netherlands [16], United Kingdom [17], Northern Ireland [22], Spain [23,25], United States [18,19], Australia [21], and two international groups [20,24] published these guidance documents between 2004 and 2014. One document specifically covered children [21], the others primarily targeted adults. Six groups reported undertaking a systematic review and appraisal of the evidence [16-20,24]. Only three were explicit about the level of evidence that underpinned their recommendations [16,18,20], and only two graded the strength of the guidance recommendations themselves [18,20]. Five guidance documents covered hyponatremia broadly; one specifically covered it in the setting of primary care, one in liver cirrhosis, one in neurosurgery, and one in exercise-associated hyponatremia. Three included treatment only [23-25], the seven others covered diagnosis as well [16-22]. Two groups reported funding by a governmental institution [16,22], one by the professional societies they represented [20]; the others did not report their funding sources [17-19,21,23-25].

**Table 1:** **Characteristics of included guidelines and consensus statements**

**Developer**	**Year**	**Country**	**Funding source**	**Target population**	**Target users**	**Guideline writers**	**Guideline review**	**Guideline update**	**Methods support**	**Evidence base**
**Europe**										
NIV	2012	Netherlands	Government funding	Adults with hyponatremia	Clinicians, Internists	Multidisciplinary internists, epidemiologist	Dutch Association of Internists (NIV), expert peer review	In case of breakthrough changes in diagnosis or treatment	PROVA – company specialized in Evidence Based Guideline Development	Systematic literature review
NHS	2011	UK	NS	Adults with hyponatremia in primary care	Primary care professionals within NHS	NS	NS	Planned in 2015	NS	Systematic literature review
GAIN*	2010	Northern Ireland	Government funding	Adults with hyponatremia	NS	Multidisciplinary anesthetists, clinical chemist, nephrologist	NS	3 years	NS	NS
AEEH*	2003-2004	Spain	NS	Patients with cirrhosis	NS	Gastroenterologists	NS	NS	NS	NS
EHN*	2013	Spain	NS	Hospitalized patients with SIADH	NS	Multidisciplinary endocrinologists, nephrologists, internists, hospital pharmacist	NS	NS	NS	Consensus statements
ERBP/ESE/ESICM	2014	Europe	Unrestricted grant from participating societies	Adults with hyponatremia	Health care professionals dealing with hyponatremia	Multidisciplinary nephrologists, endocrinologists, general internists, critical care physicians	External review by KHA-CARI, ESA, and members ERA-EDTA	5 years or earlier in case of new evidence requiring changes	ERBP methods support team	Systematic literature review
**North America**										
UF	2008-2009	USA	NS	Neurosurgery patients with hyponatremia	NS	Multidisciplinary neurosurgeons, nurse practitioners, nephrologists, critical care physician, endocrinologist, pharmacist, nurses	NS	NS	NS	Systematic literature review
HEP	2013	USA	Funding Unrestricted educational grant from pharmaceutical company	Patients with hyponatremia	NS	Endocrinologist, nephrologists	NS	NS	NS	Systematic literature review
**Australia**										
RCHM*	2012	Australia	NS	Children	NS	NS	External review within the hospital where appropriate’	12 to 24 months	NS	NS
**International**										
EAH- ICD*	2007	USA, Canada, UK, Switzer-land, Canada, South Africa, New Zealand, Australia	No commercial sponsorship’	People with exercise-associated hyponatremia	Medical personnel, athletes, greater public	Multidisciplinary endocrinologist, epidemiologist, nephrologists, emergency medicine physician, general practitioner, internist, sports physicians, exercise physiologists	NS	NS	NS	Systematic literature review

NIV, Nederlandse Internisten Vereniging [16]; NHS, National Health Service [17]; GAIN, Guidelines and Audit Implementation Network [22]; AEEH, La Asociación Española para el Estudio del Hígado [23]; EHN, European Hyponatremia Network [25]; ERBP, European Renal Best Practice; ESE, European Society of Endocrinology; ESICM, European Society of Intensive Care Medicine [20]; UF, University of Florida [18]; HEP, Hyponatremia Expert Panel [19]; RCH Melbourne, the Royal Children’s Hospital Melbourne [21]; EAH-ICD, International Exercise-Associated Hyponatremia Consensus Development Conference [24]; [Na], Serum sodium concentration; NS, Not stated; KHA-CARI, Kidney Health Australia, Caring for Australasians with Renal Impairment; ESA, Endocrine Society of Australia; ERA-EDTA, European Renal Association; European Dialysis and Transplant Association; *Classified as consensus statement.

### Appraisal of guidelines and consensus statements

**Figure 2:**
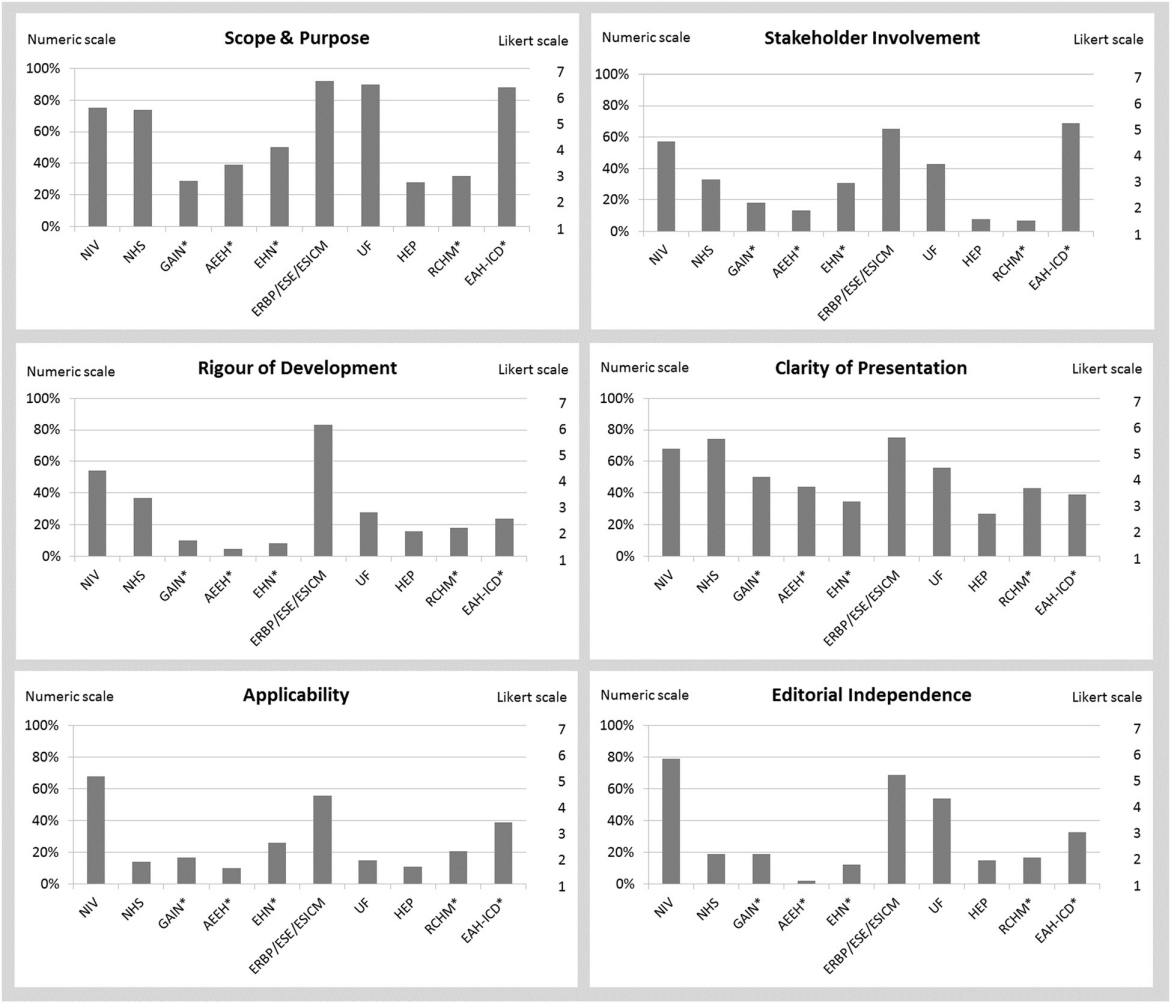
**Guideline assessment according to the appraisal of guideline for research and evaluation (AGREE II) instrument.** NIV, Nederlandse Internisten Vereniging [16]; NHS, National Health Service [17]; GAIN, Guidelines and Audit Implementation Network [22]; AEEH, La Asociación Española para el Estudio del Hígado [23]; ERBP, European Renal Best Practice; ESE, European Society of Endocrinology; ESICM, European Society of Intensive Care Medicine [20]; UF, University of Florida [18]; HEP, Hyponatremia Expert Panel [19]; RCH Melbourne, the Royal Children’s Hospital Melbourne [21]; EAH-ICD, International Exercise-Associated Hyponatremia Consensus Development Conference [15]; *Classified as consensus statement. Note: items were originally scored on a Likert scale of 1 [Strongly Disagree] to 7 [Strongly Agree]. The numerical scores presented for each domain are a summary of individual item scores by each reviewer.

Figure 2 shows the standardized domain scores for each guideline for each of the six quality domains assessed with the AGREE II tool (See Additional file 5 for mean individual scores per item across reviewers). The overall quality of reporting of the guideline development process as assessed by AGREE varied widely both between guidance documents across domains and within guidance documents between domains. Overall, guideline developers reported the details of the guideline development process only to a limited extent. Most had average scores below 50% in four to six of the six AGREE II domains [17,19,21-25], only two received an average >50% on all six [16,20].

Guidelines received the highest scores for scope and purpose (median 62%; range 28% to 92%) and clarity of presentation (median 47%; range 27% to 75%), and lowest scores for applicability (median 19%; range 10% to 68%) and editorial independence (median 19%; range 2% to 79%).

Initial appraisal results differed more than one point on the Likert scale between two or more reviewers for 143/230 items (62%). The majority of discrepancies were found in the domain ‘Clarity of Presentation’, with 90% of items differing more than one point. Group discussion resulted in 287/920 (31%) of individual entries being changed. Finally, no scores differed more than two points and for 82% of items, scores were the same or within one point of each other. Major reasons for changing an entry were a change of own opinion after clarification of the opinion of other reviewers during the group discussion (180/920 entries; 20% entries); aiming for consistency between entries given same available data (39/920; 4%); re-evaluation of the score in light of a noted comment during the appraisal process (30/920; 3%); correction for available data that were overlooked during the initial appraisal (22/920; 2%); misinterpretation of the question during the initial appraisal (6/920; 0.7%); adjusting for arbitrary scoring of items that were felt to be inapplicable for some reason (3/920; 0.3%); adjusting for inconsistent approach to deal with the assumption that a criterion was fulfilled even if this was not clearly mentioned (4/920; 0.4%); and data entry error (3/920, 0.3%). Overall, this resulted in 29/60 (48%) of standardized domain scores being downgraded by a maximum of 10% and 10/60 (17%) of standardized domain scores being upgraded with a maximum of 10%; the remaining 35% remained unchanged.

### Synthesis of recommendations

The included guidance documents addressed three major themes: diagnosis, treatment, and speed of correction.

### Approaches to diagnostic strategies for hyponatremia

Seven guidance documents covered diagnosis and differential diagnosis of hyponatremia [16-22]. Table 2 shows the key recommendations. The key areas addressed included the threshold for initiating diagnostic workup, confirmation and classification of hypotonic hyponatremia, and identification of the underlying disorder.

**Table 2:** **Summary of recommendations for approaches to diagnosis of hyponatremia by included guidance documents**

	**Guideline Organization/Society**									
**Criteria/Categories**	NIV [16]	NHS [17]	GAIN [22]	AEEH [23]	EHN [25]	ERBP/ESE/ESICM [20]	UF [18]	HEP [19]	RCHM [21]	EAH-ICD [24]
Threshold workup [Na]	<135 mmol/L	<135 mmol/L	<135 mmol/L	<130 mmol/L	<135 mmol/L	<135 mmol/L	<131 mmol/L	<135 mmol/L	<135 mmol/L	
Confirming hypotonic hyponatremia	Serum osmolality <275 mOsm/kg	Plasma osmolality <280 mOsm/kg	Serum osmolality <275 mOsm/kg		Plasma osmolality <275 mOsm/kg	Serum osmolality <275 mOsm/kg	Serum osmolality <285 mOsm/kg	Plasma osmolality <280 mOsm/kg	Serum osmolality threshold not stated	
How to classify hypotonic hyponatremia to aid identification of underlying cause										
Volume status/hydration state/extracellular fluid status	Clinical evaluation	Physical examination/clinical signs of dehydration or edema	Physical examination/clinical signs of dehydration or edema		Physical examination/clinical signs of low circulating volume	Physical examination/clinical signs of dehydration or edema	Physical examination/laboratory measurements	Physical examination/laboratory measurements	To assess but method not stated	
Urinary [Na]/Threshold	30 mmol/L	Spot urine: 20–30 mmol/L	15 mmol/L		40 mmol/L	30 mmol/L	25 mmol/L	Spot urine: 20–30 mmol/L	No threshold stated	
Urinary osmolality/Threshold	100 mOsm/kg	100 mOsm/kg	100 mOsm/kg		100 mOsm/kg	100 mOsm/kg	100 mOsm/kg	100 mOsm/kg	No threshold stated	
How to identify the underlying disorder										
History		Medications	Medications			Diuretic use				
		Fluid intake	Recently prescribed intravenous fluids							
		Nocturnal polyuria	Vomiting/diarrhea							
Lab tests										
Serum potassium concentration	+	+							+	
Serum chloride concentration		+							+	
Serum urea concentration	+/–	+					+/–	+/–	+	
Serum creatinine concentration	+	+					+/–	+/–	+	
Serum glucose concentration	+	+	+/–			+			+	
Urinary potassium concentration	+						+			
Renal tests			+							
Liver tests			+				+/–			
Urinary protein		+/–								
Thyroid function tests	+/–	+/–	+/–				+/–			
Adrenal function tests	+/–	+/–	+/–				+/–			
Serum protein electrophoresis		+/–								
Urine protein electrophoresis		+/–								
Fractional sodium excretion	+/–									
Serum uric acid concentration	+/–	+/–					+	+/–		
Fractional uric acid concentration	+/–									
Fractional excretion urea	+/–									
Urinary chloride concentration	+/–						+	+/–		
Molar weight urine	+/–									
Serum bicarbonate concentration							+/–			
Hematocrit							+/–			

[Na], Serum sodium concentration; +, always; +/–, If clinically indicated/sometimes useful.

NIV, Nederlandse Internisten Vereniging [16]; NHS, National Health Service [17]; GAIN, Guidelines and Audit Implementation Network [22]; AEEH, La Asociación Española para el Estudio del Hígado [23]; EHN, European Hyponatremia Network [25]; ERBP, European Renal Best Practice; ESE, European Society of Endocrinology; ESICM, European Society of Intensive Care Medicine [20]; UF, University of Florida [18]; HEP, Hyponatremia Expert Panel [19]; RCH Melbourne, the Royal Children’s Hospital Melbourne [21]; EAH-ICD, International Exercise-Associated Hyponatremia Consensus Development Conference [24].

Guidance documents differed somewhat in their recommended threshold for starting diagnostic assessment. Six recommended starting diagnostic assessment when the serum sodium concentration dropped below 135 mmol/L [17,19-23] and to confirm hypotonicity through a measured serum or plasma osmolality <275 to 285 mOsm/kg [16-20,22]. Two others set lower thresholds of serum sodium concentration at <131 mmol/L [18] and <130 mmol/L [23]. Six guidance documents advised classifying hypotonic hyponatremia into categories of hypovolemia, euvolemia, and hypervolemia to aid differential diagnosis and guide treatment [16-22]. Most guidance documents recommended a clinical assessment of hydration status and a urinary sodium concentration as well as a urinary osmolality measurement, although specific criteria, thresholds, and algorithms differed.

Most guidance documents proposed additional laboratory tests that could be of value to identify the underlying disorder, but they varied substantially regarding which tests to use in what situation and which reference values to use. Only two explicitly recommended taking a history of drug intake and symptoms as part of the assessment [17,22]. Four presented an algorithm to guide differential diagnosis [16,18,20,22].

### Approaches to treatment for hyponatremia

Table 3 shows the recommendations for the medical management of hyponatremia. Guidance documents distinguished treatment scenarios based on whether patients had severe symptoms [17-22,24,25] or whether the hyponatremia was acute (48 h) or chronic [16]. All but one discussed treatment in the setting of severe symptoms and recommended infusion of hypertonic saline, usually specified as having a concentration of 3% [17,19-21,24,25]. One suggested using a formula to guide the infusion speed of a continuous infusion [16], five others recommended giving a fixed dose [19,20,22,24,25], or a dose adjusted to body weight [21,25] with repeated serum sodium concentration measurements to check progression [16,20-22,25].

**Table 3:** **Summary of recommendations for approaches to treatments for hyponatremia by included guidance documents**

	**Guideline Organization/Societies**									
**Criteria/categories**	NIV [16]	NHS [17]	GAIN [22]	AEEH [23]	EHN [25]	ERBP/ESE/ESICM [20]	UF [18]	HEP [19]	RCHM [21]	EAH-ICD [24]
**Symptoms**										
Acute Onset (<48 h)	NaCl >1% Infusion speed may be guided by Adrogué-Madias	NaCl 3%	NaCl 2.7% 200 mL over 30 min		NaCl 3% 100 mL/10 min up to 3× or infused at 0.5–2 mL/kg/h	NaCl 3% 150 mL/20 min up to 4×	NaCl >1%	NaCl 3% 100 mL/10 min up to 3× or infused at 0.5–2 mL/kg/h	NaCl 3% 4 mL/kg over 30 min	NaCl 3% 100 mL bolus
*Hypovolemia*								NaCl 0.9% until blood pressure restored		
*Euvolemia*			Fluid restriction							No hypotonic fluids
			Stop offending drugs							
			Stop hypotonic fluids							
*Hypervolemia*			Furosemide					Furosemide		
Chronic onset (>48 h)	NaCl >1% Infusion speed calculation may be guided by Adrogué-Madias	NaCl 3%	Only if severe symptoms NaCl 2.7% 200 mL over 30 min infusion speed by may be guided Adrogué-Madias		NaCl 3% 100 mL/10 min up to 3× or infused at 0.5–2 mL/kg/h	NaCl 3% 150 mL/20 min up to 4×	NaCl >1%	NaCl 3% 100 mL/10 min up to 3× or infused at 0.5-2 mL/kg/h		
*Hypovolemia*			NaCl 0.9% 1 L over 2–4 h infusion speed may be guided by Adrogué-Madias					NaCl 0.9% until blood pressure restored		
*Euvolemia*			Fluid restriction							
			Stop offending medications							
			Stop hypotonic fluids							
*Hypervolemia*			Fluid restriction					Furosemide		
			Salt restriction							
**No symptoms**										
Acute onset (<48 h)	NaCl >1% Infusion speed by Adrogué-Madias		Treat underlying condition			Stop offending fluids and medications, treat underlying condition NaCl 3% 150 mL/20 min		Treat underlying condition		
Chronic onset (>48 h)	Treat underlying condition		Treat underlying condition			Stop non-essential fluids Stop offending medications Treat underlying condition		Treat underlying condition		
*Hypovolemia*	NaCl 0.9%	NaCl 0.9% until blood pressure restored	NaCl 0.9% infusion speed may be guided by Adrogué-Madias			NaCl 0.9% or balanced crystalloid 0.5–1 mL/kg/h	NaCl 0.9%	NaCl 0.9% until blood pressure restored	Nasogastric rehydration	
	NaCl tablets							No VPA	NaCl 0.9%	
*Euvolemia*	Fluid restriction, dose dependent on serum and urinary electrolytes	Fluid restriction, 500–1,000 mL/d	Fluid restriction		Fluid restriction <500–1,000 mL/d	Fluid restriction	Fluid restriction	Fluid restriction 500 mL below average daily urine output	Fluid restriction, no hypotonic fluids	
		No salt restriction	Salt restriction		Salt 5–8 g/d			No salt restriction		
	Loop diuretics				Furosemide 20–60 mg/d + oral NaCl	Loop diuretics, low dose + oral NaCl	Diuretics			
	Demeclocycline	Demeclocycline				No demeclocycline	Demeclocycline	Demeclocycline, 600–1,200 mg/d		
	Urea				Urea 30 g/d	Urea, 0.25–0.5 g/kg/d	Urea	Urea, 15–60 g/d		
	Vasopressin receptor antagonist	Vasopressin receptor antagonist			Tolvaptan 15–60 mg/d	No vasopressin receptor antagonists				
*Hypervolemia*		Treat underlying condition								
	Fluid restriction, dose dependent on serum and urinary electrolytes	Fluid restriction	Fluid restriction	Fluid restriction <1,000 mL/d		Fluid restriction		Fluid restriction, <insensible losses + urine output	Fluid restriction	
	Loop diuretics	Salt restriction	Salt restriction	No NaCl >0.9%				Salt restriction		
		Demeclocycline		Stop diuretics		No demeclocycline		Possibly vasopressin receptor antagonist		
		Vasopressin receptor antagonist				No vasopressin receptor antagonist				

NIV, Nederlandse Internisten Vereniging [16]; NHS, National Health Service [17]; GAIN, Guidelines and Audit Implementation Network [22]; AEEH, La Asociación Española para el Estudio del Hígado [23]; EHN, European Hyponatremia Network [25]; ERBP, European Renal Best Practice; ESE, European Society of Endocrinology; ESICM, European Society of Intensive Care Medicine [20]; UF, University of Florida [18]; HEP, Hyponatremia Expert Panel [19]; RCH Melbourne, the Royal Children’s Hospital Melbourne [21]; EAH-ICD, International Exercise-Associated Hyponatremia Consensus Development Conference [24].

Patients without symptoms of hyponatremia were assumed to have chronic onset hyponatremia, and treatment suggestions were mostly dependent on the classification hypovolemic, euvolemic, or hypervolemic. Only three guidance documents specifically advised treating the underlying condition [19,22]. Seven suggested 0.9% saline in hypovolemia [16-22], with infusion speeds calculated with Adrogué-Madias [22], until restoration of blood pressure [17,19] or until nasogastric rehydration could start [21].

For euvolemic asymptomatic hyponatremia, the majority recommended fluid restriction as the first-line treatment [16-25]. Five guidance documents proposed a number of other interventions as second-line treatments including loop diuretics [16,18,20,25], demeclocycline [16-19], urea [16,19,20,25], vasopressin receptor antagonists [16,17,25], or lithium [18]. One guideline specifically recommended against vasopressin receptor antagonists in case of a serum sodium concentration <125 mmol/L [20].

For hypervolemic asymptomatic hyponatremia, seven guidance documents recommended fluid restriction as the first-line treatment [16,17,19-23] (Table 3). Three guidance documents advocated concomitant salt restriction, without clear dose recommendations [17,19,22], and one to avoid hypotonic infusion solution [21]. Three additionally proposed loop diuretics [16,17,19] and three others generally stated to treat the underlying disease [17,20,22], whereas one advised to consider stopping diuretics [23]. One guideline additionally proposed demeclocycline and two proposed vasopressin receptor antagonists as a second-line treatment for refractory hyponatremia [17,19], whereas one guideline specifically recommended against both demeclocycline and vasopressin receptor antagonists [20].

### Targets and limits of speed of correction

Table 4 shows the key recommendations. The key areas include targets and limits for increase in serum sodium concentration.

**Table 4:** **Summary of recommendations for targets and limits for speed of correction of hyponatremia by included guidance documents**

	**Guideline Organization/Societies**									
**Criteria/categories**	NIV	NHS	GAIN	AEEH	EHN	ERBP/ESE/ESICM	UF	HEP	RCHM	EAH-ICD
	[16]	[17]	[22]	[23]	[25]	[20]	[18]	[19]	[21]	[24]
**Targets [Na] increase**										
Symptoms	Independent of symptoms	If symptoms	If symptoms		If symptoms	If symptoms		If symptoms	Until seizures resolve or [Na] >125 mmol/L	
Acute onset (<48 h)	1–2 mmol/L/h initially	Until [Na] >120 mmol/L independent of onset	1–2 mmol/L/h first 2–3 h		1–6 mmol/L first 2 h	5 mmol/L first h		4–6 mmol/L urgently	Independent of onset	
Chronic onset (>48 h)			0.5–1 mmol/L/h first 2–3 h		1–6 mmol/L first 2 h	5 mmol/L first h		If seizures or coma 4–6 mmol/L urgently, otherwise 4–6 mmol/L per 24 h		
**Limits [Na] increase**										
Symptoms	Independent of symptoms	If no symptoms	Independent of symptoms		Independent of symptoms	Independent of symptoms	Independent of symptoms	If no symptoms	Symptom dependent	
Acute onset (<48 h)	If no risk of ODS ≤10 mmol/L per 24 h ≤18 mmol/L per 48 h If risk of ODS <8 mmol/L per 24 h	≤8–12 mmol/L per 24 h ≤18 mmol/L per 48 h	<12 mmol/L per 24 h		If no risk of ODS ≤10 mmol/L per 24 h ≤18 mmol/L per 48 h If risk of ODS <8 mmol/L per 24 h	≤10 mmol/L first 24 h ≤8 mmol/L every 24 h thereafter	≤10 mmol/L per 24 h	No limits	≤8 mmol/L per 24 h after seizures resolve, Independent of onset	
Chronic onset (>48 h)	<8 mmol/L per 24 h	≤8–12 mmol/L per 24 h ≤18 mmol/L per 48 h	<12 mmol/L per 24 h		<8–12 mmol/L per 24 h <18 mmol/L per 48 h	≤10 mmol/L first 24 h ≤8 mmol/L every 24 h thereafter	≤10 mmol/L per 24 h	<8–12 mmol/L per 24 h <18 mmol/L per 48 h		

[Na] – Serum sodium concentration.

NIV, Nederlandse Internisten Vereniging [16]; NHS, National Health Service [17]; GAIN, Guidelines and Audit Implementation Network [22]; AEEH, La Asociación Española para el Estudio del Hígado [23]; EHN, European Hyponatremia Network [25]; ERBP, European Renal Best Practice; ESE, European Society of Endocrinology; ESICM, European Society of Intensive Care Medicine [20]; UF, University of Florida [18]; HEP, Hyponatremia Expert Panel [19]; RCH Melbourne, the Royal Children’s Hospital Melbourne [21]; EAH-ICD, International Exercise-Associated Hyponatremia Consensus Development Conference [24].

Seven guidance documents provided targets or aims for the increase in serum sodium concentration in case of symptomatic and/or acute hyponatremia [16,17,19-22,25]. Seven guidance documents provided limits for the increase in serum sodium concentration that should not be surpassed [16-22,25]. Five did so independent of symptoms [16,18,20,22,25]. Limits usually varied between 8 to 12 mmol/L during the first 24 hours [16-22,25] and 18 mmol/L during the first 48 hours [16,17,19,20,25], irrespective of whether hyponatremia was acute or chronic [16,17,20,25]. Three guidance documents set a stricter limit of <8 mmol/L during the first 24 hours in cases where the patient was believed to be high risk for developing osmotic demyelination syndrome [16,19,25]. Four discussed what to do in case of overcorrection, i.e., to stop current treatment and to consider re-lowering serum sodium concentration by starting hypotonic infusion and administering 1 to 4 μg desmopressin every 6 to 8 hours [16,19,20,25].

## Discussion

We found five clinical practice guidelines and five consensus statements covering the diagnostic approach to and treatment of hyponatremia. Although most used serum osmolality, volume status, urinary sodium, and urinary osmolality to guide differential diagnosis, they differed in classification thresholds, what additional tests to consider, and when to initiate diagnostic work-up. Most advocated hypertonic NaCl in severely symptomatic, acute onset hyponatremia and NaCl 0.9%, fluid restriction, and cause-specific therapy for hypovolemic, euvolemic, and hypervolemic hyponatremia, respectively. However, they somewhat differed in the limits for speed of increase in serum sodium concentration and which specific medications to use. The reasons for offering different recommendations are undoubtedly multifactorial. They may in part be explained by the fact that recommendations were issued by organizations differing in context and scope. It is also very likely that some variability in guidance arose through limitations in the evidence available for guideline developers to base their recommendations on [8]. In the most recent guideline on diagnosis and treatment of hyponatremia, 98% of the graded recommendations were based on very low and low level of evidence, while none were based on a high level of evidence. The lack of high quality evidence may have increased the part opinion had to play in framing the recommendations. In addition, the evidence that was available may have been interpreted differently dependent on the importance for decision making given to certain outcomes (e.g., serum sodium concentration). Finally, differences in personal experience due to differing availability of medications may partly explain possible differences in perception of uncertainties around drug safety.

However, it is also possible that discrepancies between guidance documents may in part be explained by differences in underlying methods of development. Quality, as assessed by AGREE II, was suboptimal at best, with only two documents obtaining a score >50% for each of the six quality domains [16,20]. The findings suggest that several aspects related to methodological rigor of development, stakeholder involvement, applicability, and editorial independence could be improved, possibly improving consistency in provided guidance. This is in line with the findings of a recent overview of 42 appraisal studies including a total of 626 clinical practice guidelines across several clinical disciplines [26]. For guidelines to be trustworthy, they must be i) founded on high quality systematic reviews, ii) include the relevant stakeholders, and iii) be applicable in clinical practice [9].

Only half of the guidance groups stated they had conducted a systematic review of the evidence. Save one, the reviews would not have met the Institute of Medicine’s criteria for reporting high-quality systematic reviews [20,27], because key methods for finding and assessing individual studies as well as synthesizing the body of evidence were not described. Conducting high-quality systematic reviews requires specific methodological expertise and support which may not be available to most groups [27]. One solution might be to harmonize effort across organizations, thus focusing resources, allowing higher quality reviews and reducing duplication and possibly inconsistency between guidelines.

Six groups included healthcare professionals from different specialties [16,18,20,22,24,25]. Multidisciplinary contribution serves to broaden the approach to health-care problems, increase the completeness of evidence-finding strategies, and help to identify hurdles to implementation. When reflecting on approaches to hyponatremia, bringing together several disciplines mirrors the clinical reality of multiple specialty areas dealing with the same problem but looking at it from a different angle. Only one of the development groups reported considering patients’ views and experiences, but even then did so to a limited extent [20]. Decisions on clinical care should factor in patient values and preferences. Interventions for chronic hyponatremia, such as fluid restriction, may affect quality of life and patient preference should influence the ultimate recommendations.

Low scores for applicability mostly reflect the absence of describing barriers to guideline implementation and failure to provide tools for putting the recommendations into practice. In part, guidelines are designed to deal with the challenges of increasing knowledge and time-pressure. They are designed to help make decisions at the point of care. However, being often lengthy publications without layered presentation of information, it is likely that the majority of the guidance documents may not reach their target audience or stimulate implementation. Four guidance documents provided algorithms for diagnosis or treatment [16,18,20,25]; although these are likely to increase the utility of a guideline, it is unclear to what extent they truly improve implementation of the recommendations. How to best communicate evidence-based recommendations to the relevant stakeholders is a recent but active area of research lead by the DECIDE consortium [28]. With results of their research expected, guideline developers will have additional targets for improving the applicability in the future.

To our knowledge, this is the first attempt to systematically synthesize and appraise clinical guidelines on the diagnosis and treatment of hyponatremia In accordance with the Prisma statement, we conducted a comprehensive literature search and searched an additional 337 websites of specialist societies and guideline organizations [29] (Additional file 6). We used AGREE II, a validated and reliable instrument, and an adequate number of reviewers to individually appraise the guidance documents [30]. On top of the individual appraisals, we included an attempt to resolve major discrepancies and increase consistency by introducing an audiotaped group consensus meeting. During this meeting, reviewers could explain and motivate their scores and adapt them if they wanted to. This mostly resulted only in modest downgrading of domain scores by 1% to 10%. Most of the changes happened because reviewers felt they had scored inconsistently for a same rationale, or because they missed information during the initial appraisal that was in fact available in the document. Although the scores did not change substantially, the group felt the discussion further highlighted the qualitative differences between the guidance documents. In addition, even the reviewers with large deviations from the mean in their initial scores felt they agreed with the conclusion. It means that final average scores were truly a product of consensus rather than a mathematical calculation, as proposed in the original AGREE protocol. We believe that a consensus meeting is valuable in any guideline appraisal process, and particularly useful if reviewer groups have the intention to select a guideline for local use.

This study has its limitations. We based our assessment on what guideline organizations actually reported. Reporting by guideline developers may not wholly reflect what occurred in practice with respect to the AGREE criteria, and we did not seek additional clarification. However, contacting guideline developers is not standard practice when using AGREE as the instrument specifically aims to provide a framework for assessing the quality of reporting of recommendations. We aimed to summarize the existing recommendations on diagnosis and treatment of hyponatremia as formulated by other guideline development groups and to evaluate the quality of the guideline development process. We did not aim to summarize or critically appraise the evidence base itself. Consequently, it is difficult to assess to what extent differences between guidance documents stem from differences in development procedures rather than important limitations in the evidence base that underpin individual recommendations. Secondly, the purpose of using the AGREE instrument was not to accuse guideline development groups of being biased, but rather to highlight both strengths and weaknesses of existing guidance to suggest on how we could make improvements in the future.

Calculation of summary scores for each domain across reviewers required summing up all the scores of the individual items in a domain for each reviewer and then standardizing this total as a percentage of the maximum possible score for that domain. In doing so, the originally semi-qualitative Likert scale was converted to a quantitative score. This may have introduced numeric differences between the guidance documents that were beyond the discriminatory ability of the tool and possibly negligible in practice. Finally, we acknowledge that four of the authors of this paper also authored one of the guidelines included in this review. Although we aimed to judge all guidance documents fairly against the criteria outlined by the AGREE instrument, we cannot rule out that a subconscious intellectual competing interest unduly influenced the scoring.

## Conclusions

Current guidelines on the assessment and treatment of hyponatremia often fail to meet methodological criteria for development and reporting as described by AGREE II. Despite many similarities, recommendations are sometimes inconsistent, but to what extent this is attributable to the underlying development process remains unclear.

## Additional files

Additional file 1: Table S1. Search strategies. Additional file 2: Table S2. Data extraction template. Additional file 3: Table S3. Structure and content of the AGREE instrument. Additional file 4: Table S4. Table of excluded studies. Additional file 5: Table S5. Mean scores across reviewers for the individual AGREE II domain items. Additional file 6:
**PRISMA checklist.**

## Abbreviation

AGREE II: Appraisal of guidelines for research and evaluation II
