# Supplementary material for: Diagnosis and treatment of hyponatremia: a systematic review of clinical practice guidelines and consensus statements
Source: BMC Med. 2014 Dec 11;12:1. doi: 10.1186/s12916-014-0231-1 (PMC4276109; doi:10.1186/s12916-014-0231-1)
Supplement: Additional file 1: Table S1. — Search strategies. [file 12916_2014_231_MOESM1_ESM.pdf]

Additional file 1: Table S1. Search Strategies

Search Strategy MEDLINE

1. Hyponatremia/
2. Inappropriate ADH Syndrome/
3. Water Intoxication/
4. hyponatr?emia.tw.
5. SIAD$.tw.
6. or/1-5
7. exp guideline/
8. consensus/
9. exp consensus development conference/
10. consensus.tw.
11. position statement$.tw.
12. exp Policy/
13. recommendation$.tw.
14. or/7-13
15. 6 and 14

Search Strategy EMBASE

1. Hyponatremia/
2. Inappropriate Vasopressin Secretion/
3. Water Intoxication/
4. hyponatr?emia.tw.
5. SIAD$.tw.
6. or/1-5
7. exp practice guideline/
8. guideline$.tw.
9. consensus.tw.
10. position statement$.tw.
11. exp health care policy/ or exp policy/
12. recommendation$.tw.
13. or/7-12
14. 6 and 13

Additional Searches

We additionally conducted an online search for professional societies and organisations within the fields of Internal Medicine, Nephrology, Endocrinology and Intensive Care Medicine to identify published guidelines or consensus statements.

These searches were conducted with the Google Chrome web browser using the following strategy:

We first tried to find international societies for each of the disciplines and searched their member societies if a website was provided. If a website was not provided we entered the provided name of the society in the web browser and scanned the first 10 citations. In addition we retrieved a list of the countries of the world from https://www.cia.gov/library/publications/the-world-factbook/index.html (accessed on 02/04/2013) and entered each name on the list in combination with the field name and the words 'society' and 'association' and scanned the first 10 citations. An example of the search:

Belgium AND (Nephrology) AND (Society OR Association)

We searched the website of the identified societies, if a uniform resource locator was identified. If a website included a search utility, we searched for guidelines and consensus statements on hyponatraemia by sequentially entering the

search terms ‘hyponatraemia’ and ‘hyponatremia’. If the language used on the website was not English, we entered a search term for hyponatraemia in the appropriate language. We screened all citations thus retrieved. If a website did not provide a search function, we manually screened the website to identify guidance documents.

Finally, we conducted a ‘liberal’ search of the internet using the strategy (hyponatraemia OR hyponatraemia) AND Guideline and screened the first 100 citations.

The included table below shows the identified societies, a uniform resource locator, the date on which the site was accessed, the search strategy used and the number of citations retrieved and the number of guidance documents withheld. EN conducted all additional searches. EN and JV examined all full-texts thus withheld for eligibility.

Table Additional Searches

	Field	Country	Society/ Organisation	National Name	URL	Date Last Search	Search Strategy	Titles	CPG	CPG not devoted to hypo-natraemia
**1**	Guidelines	International	National Guideline Clearinghouse		http://www.guideline.gov/	20/12/2013	Hyponatreamia or Hyponatraemia	38	0	9
**2**	Guidelines	International	Guidelines International Network		www.g-i-n.net	20/12/2013	Hyponatreamia or Hyponatraemia	1	0	0
**3**	Guidelines	International	NHS Evidence		https://www.**evidence**.**nhs**.uk	20/12/2013	Hyponatreamia or Hyponatraemia	436	0	0
**4**	Guidelines	International	Centre for Reviews and Dissemination		www.crd.york.ac.uk	20/12/2013	Hyponatreamia or Hyponatraemia	12	0	0
**5**	Guidelines	International	NICE		www.**nice**.org.uk	20/12/2013	Hyponatreamia or Hyponatraemia	4	0	0
**6**	Guidelines	United Kingdom	Guidelines and Audit Implementation Network		http://www.gain-ni.org	20/12/2013	hyponatraemia/hyponatremia	81	1	
**7**	Guidelines	United Kingdom	National Institute for Health and Care Excellence Clinical Knowledge Summaries		http://cks.nice.org.uk/	20/12/2013	hyponatraemia/hyponatremia	4	1	
**8**	Internal Medicine	Argentina	Argentine Medical Association	Asociación Médica Argentina	http://www.ama-med.org.ar/	20/12/2013	hiponatremia/hyponatremia/hyponatraemia	0		
**9**	Internal Medicine	Armenia	Armenian Medical Society		http://armeda.am/eng/page/about-us/	20/12/2013	hyponatraemia/hyponatremia	0		
**10**	Internal Medicine	Australia/New Zealand	Australia & New Zealand Royal College of Physicians		www.racp.edu.au	20/12/2013	hyponatraemia/hyponatremia	0		
**11**	Internal Medicine	Austria	Austrian Society of Internal Medicine		www.oegim.at	20/12/2013	Hyponatriämie/hyponatremia/hyponatraemia	0		
**12**	Internal Medicine	Bangladesh	Bangladesh Society of Internal Medicine			20/12/2013				
**13**	Internal Medicine	Belarus	Society of Internal Medicine of Belarus			20/12/2013				
**14**	Internal Medicine	Belgium	Belgian Society of Internal Medicine	Société Belge de Médecine Interne	www.bsim.be	20/12/2013	hyponatraemia/hyponatremia	3	0	
**15**	Internal Medicine	Bolivia	Bolivian Society of Internal Medicine			20/12/2013				

	Field	Country	Society/ Organisation	National Name	URL	Date Last Search	Search Strategy	Titles	CPG	CPG not devoted to hypo-natraemia
**16**	Internal Medicine	Brazil	Brazilian Society of Internal Medicine	Sociedade Brasileira de Clinica Medica	www.sbcm.org.br	20/12/2013				
**17**	Internal Medicine	Brunei	Brunei Medical Association		na	20/12/2013				
**18**	Internal Medicine	Bulgaria	Bulgarian Society of Internal Medicine			20/12/2013				
**19**	Internal Medicine	Canada	Canadian Society of Internal Medicine		http://www.csim.ca/index.php/en/	20/12/2013	hyponatraemia/hyponatremia	1	0	
**20**	Internal Medicine	Chile	Chilean Society of Internal Medicine	Sociedad Chilena de Medicina Interna	www.smschile.cl	20/12/2013	hiponatremia/hyponatremia/hyponatraemia	0		
**21**	Internal Medicine	China	Chinese Society of Internal Medicine,			20/12/2013				
**22**	Internal Medicine	Colombia	Colombian Society of Internal Medicine	Asociacion Colombiana de Medicina Interna	www.acmi.org.co	20/12/2013	hiponatremia/hyponatremia/hyponatraemia	0		
**23**	Internal Medicine	Croatia	Croatian Society of Internal Medicine			20/12/2013				
**24**	Internal Medicine	Cuba	Cuban Society of Internal Medicine	Sociedad Cubana de Medicina Interna		20/12/2013				
**25**	Internal Medicine	Czech Republic	Czech Society of Internal Medicine		www.interna-cz-eu	20/12/2013				
**26**	Internal Medicine	Dominican Republic	Society of Internal Medicine of the Dominican Republic	Sociedad de Medicina Interna de la República Dominicana (SMIRD)		20/12/2013				
**27**	Internal Medicine	Egypt	Egyptian Society of Internal Medicine		http://www.esim.org.eg/	20/12/2013				
**28**	Internal Medicine	Equador	Equatorian Society of Internal Medicine	Asociacion Ecuatoriana de Medicina Interna	aemi.med.ec	20/12/2013				
**29**	Internal Medicine	Estonia	Estonian Society of Internal Medicine			20/12/2013				
**30**	Internal Medicine	Finland	Finnish Society of Internal Medicine			20/12/2013				
**31**	Internal Medicine	France	French Society of Internal Medicine	Société Nationale Française de Médecine Interne	www.snfmi.org	20/12/2013	hyponatrémie/hyponatremia/hyponatraemia	29	0	

	Field	Country	Society/ Organisation	National Name	URL	Date Last Search	Search Strategy	Titles	CPG	CPG not devoted to hypo-natraemia
32	Internal Medicine	Georgia	Georgian Society of Internal Medicine			20/12/2013				
33	Internal Medicine	Germany	German Society of Internal Medicine	Deutsche Gesellschaft für Innere Medizin	www.dgim.de	20/12/2013				
34	Internal Medicine	Ghana	Ghana Society of Internal Medecine			20/12/2013				
35	Internal Medicine	Guatemala	Guatemalan Society of Internal Medicine	Asociacion de Medicina Interna de Guatemala	www.asomigua.com	20/12/2013				
36	Internal Medicine	Hungary	Hungarian Society of Internal Medicine			20/12/2013				
37	Internal Medicine	India	Association of Physicians of India		www.apiindia.org	20/12/2013				
38	Internal Medicine	Indonesia	Indonesian Society of Internal Medicine		www.pbpapdi.org	20/12/2013	hiponatremia/hyponatremia/hyponatraemia	0	0	
39	Internal Medicine	International	International Society of Internal Medicine		http://www.isim-online.org/	20/12/2013				
40	Internal Medicine	International	International Association of Internists		http://www.iainternalmeds.com/	20/12/2013				
41	Internal Medicine	International	European Federation of Internal Medicine		http://www.efim.org/	20/12/2013	hyponatraemia/hyponatremia	0		
42	Internal Medicine	Iran	Iranian Society of Internal Medicine		www.dakheli.org	20/12/2013				
43	Internal Medicine	Israel	Israel Society of Internal Medicine			20/12/2013				
44	Internal Medicine	Italy	Italian Society of Internal Medicine		www.simi.it	20/12/2013		10	0	
45	Internal Medicine	Japan	The Japanese Society of Internal Medicine		www.naika.or.jp	20/12/2013				
46	Internal Medicine	Jordan	Jordanian Society of Internal Medicine		http://www.internalmedicine-jo.com/	20/12/2013	hyponatraemia/hyponatremia	0		
47	Internal Medicine	Korea, South	The Korean Association of Internal Medicine		www.kaim.or.kr	20/12/2013				
48	Internal Medicine	Latvia	Latvian Society of Internal Medicine			20/12/2013				
49	Internal Medicine	Lithuania	Lithuanian Society of Internal Medicine			20/12/2013				

	Field	Country	Society/ Organisation	National Name	URL	Date Last Search	Search Strategy	Titles	CPG	CPG not devoted to hypo-natraemia
**50**	Internal Medicine	Macedonia	Macedonian Assotiation of Internal Medicine		na	20/12/2013				
**51**	Internal Medicine	Mexico	Mexican Society of Internal Medicine	Asociacion de Medicina Interna de Mexico	www.cmim.org	20/12/2013				
**52**	Internal Medicine	Nicaragua	Nicaraguan Society of Internal Medicine	Asociacion Nigaragüense de Medicina Interna	www.amnica.org	20/12/2013				
**53**	Internal Medicine	Nigeria	Association of Physicians of Nigeria			20/12/2013				
**54**	Internal Medicine	Norway	Norwegian Society of Internal Medicine			20/12/2013				
**55**	Internal Medicine	Panama	Panama Society of Internal Medicine	Sociedad Panamena de Medicina Interna		20/12/2013				
**56**	Internal Medicine	Paraguay	Paraguay Society of Internal Medicine	Sociedad Paraguaya de Medicina Interna		20/12/2013				
**57**	Internal Medicine	Peru	Peruan Society of Internal Medicine	Sociedad Peruana de Medicina Interna	www.medicinainterna.com.pe	20/12/2013				
**58**	Internal Medicine	Philippines	Philippinian College of Physicians		www.pcp.org.ph	20/12/2013				
**59**	Internal Medicine	Poland	Polish Society of Internal Medicine			20/12/2013				
**60**	Internal Medicine	Portugal	Portugese Society of Internal Medicine	Sociedad Portuguesa de Medicina Interna	www.spmi.pt	20/12/2013				
**61**	Internal Medicine	Romania	Romanian Society of Internal Medicine	Rumänische Gesellschaft für Innere Medizin	www.srmi.ro	20/12/2013				
**62**	Internal Medicine	Russia	Russian Society of Internal Medicine		www.rsmsim.ru	20/12/2013				
**63**	Internal Medicine	Serbia	Serbian Association of Internal Medicine		www.uis.org.rs	20/12/2013				
**64**	Internal Medicine	Singapore	College of Physicians, Singapore		www.cps.ams.edu.sg	20/12/2013				
**65**	Internal Medicine	Slovakia	Slovak Society of Internal Medicine			20/12/2013				
**66**	Internal Medicine	Slovenia	Slovenian Association of Internal Medicine			20/12/2013				

	Field	Country	Society/ Organisation	National Name	URL	Date Last Search	Search Strategy	Titles	CPG	CPG not devoted to hypo-natraemia
**67**	Internal Medicine	South Africa	The Faculty of Consulting Physicians of South Africa		www.physician.co.za	20/12/2013				
**68**	Internal Medicine	Spain	Spanish Society of Internal Medicine	Sociedad Espãnola de Medicina Interna	www.fesemi.org	20/12/2013				
**69**	Internal Medicine	Sweden	Swedish Society of Internal Medicine		www.sim.nu	20/12/2013				
**70**	Internal Medicine	Switzerland	Swiss Society of Internal Medicine		www.sgim.ch	20/12/2013				
**71**	Internal Medicine	Taiwan	Taiwan Society of Internal Medicine		www.sim.org.tw	20/12/2013				
**72**	Internal Medicine	The Netherlands	The Netherlands Association of Internal Medicine		www.internisten.nl	20/12/2013	hyponatriëmie/hyponatremia/hyponatraemia	3	0	
**73**	Internal Medicine	Turkey	Turkish Society of Internal Medicine		www.tihud.org.tr	20/12/2013				
**74**	Internal Medicine	Ukraine	Ukrainian Association of Internal Medicine			20/12/2013				
**75**	Internal Medicine	United Kingdom	Royal College of Physicians		www.rcplondon.ac.uk	20/12/2013	hyponatraemia/hyponatremia	1	0	
**76**	Internal Medicine	Uruguay	Uruguayan Society of Internal Medicine	Sociedad de Medicina del Uruguay	www.medcinainterna.org.uy	20/12/2013				
**77**	Internal Medicine	USA	The American College of Physicians – American Society of Internal Medicine		www.acponline.org	20/12/2013				
**78**	Internal Medicine	Venezuela	Venezuelan Society of Internal Medicine	Sociedad Venezolana de Medicina Interna	www.svmi.web.ve	20/12/2013				
**79**	Nephrology	Albania	Albanian Society of Nephrology		na	21/12/2013		0		
**80**	Nephrology	Algeria	Algerian Society of Nephrology, Dialysis and Transplantation	Société Algérienne de Néphrologie, Dialyse & Transplantation	na	21/12/2013		0		
**81**	Nephrology	Argentina	Argentinian Society of Nephrology	Sociedad Argentina de Nefrologia	www.san.org.ar	21/12/2013	hiponatremia/hyponatremia/hyponatraemia	0		
**82**	Nephrology	Australia/New Zealand	Kidney Health Australia/Caring for Australasians with Renal Disease		http://www.cari.org.au	21/12/2013	hyponatraemia/hyponatremia	0		

	Field	Country	Society/ Organisation	National Name	URL	Date Last Search	Search Strategy	Titles	CPG	CPG not devoted to hypo-natraemia
83	Nephrology	Austria	Austrian Society of Nephrology	Österreichische Gesellschaft für Nephrologie	http://www.niere-hochdruck.at/	21/12/2013	Hyponatriämie	0		
84	Nephrology	Bangladesh	Bangladesh Renal Association		http://www.bra-bd.org	21/12/2013	hyponatraemia/hyponatremia			
85	Nephrology	Belarus	Bielorussian Society of Nephrology		na	21/12/2013				
86	Nephrology	Belgium	Belgian Society of Nephrolgy	Société Belge de Néphrologie - Belgische Vereniging voor Nefrologie	http://www.bvn-sbn.be	21/12/2013	hyponatriëmie	0		
87	Nephrology	Bolivia	Biolivian Society of Nephrology	Sociedad Boliviana de Nefrologia	na	21/12/2013				
88	Nephrology	Bosnia and Herzegovina	Society of Nephrology, Dialysis and Kidney Transplantation in Bosnia and Herzegovina	Udruženje ljekara za Nefrologiju, Dijalizu i Transplantaciuju Bubrega u Bosni i Hercegovini	http://www.undt.ba	21/12/2013	hyponatremia/hyponatraemia	0		
89	Nephrology	Brazil	Brazilian Society of Nephrology	Sociedade Brasileira de Nefrologia	http://www.sbn.org.br	21/12/2013	hiponatremia/hyponatremia/hyponatraemia	45	0	
90	Nephrology	Bulgaria	Bulgarian Society of Nephrology	Българското нефрологично дружество	http://www.bgnephrology.com	21/12/2013	хипонатриемия/hyponatremia/hyponatraemi a	2	0	
91	Nephrology	Canada	Canadian Society of Nephrology	Canadian Society of Nephrology	https://www.csnscn.ca/en/	21/12/2013	hyponatremia/hyponatraemia	0		
92	Nephrology	Chile	Chilean Society of Nephrology	Sociedad Chilena de Nefrologia	http://www.nefro.cl/	21/12/2013	hiponatremia/hyponatremia/hyponatraemia	0		
93	Nephrology	China	Chinese Society of Nephrology		na	21/12/2013				
94	Nephrology	Colombia	Colombian Association of Nephrology and Arterial Hypertension	Asociacion Colombiana de Nephrologia e Hipertension Arterial	http://www.asocolnef.com	21/12/2013	hiponatremia/hyponatremia/hyponatraemia	0		
95	Nephrology	Costa Rica	Costa Rican Association of Nephrology	Asociacion Costarricense de Nefrologia	http://www.ascone.org	21/12/2013				
96	Nephrology	Croatia	Croatian Society for Nephrology, Dialysis and Transplantation	Hrvatsko Društvo za Nefrologiju, Dijalizu i Transplantaciju	http://www.hdndt.org	21/12/2013	hiponatrijemija/hyponatremia/hyponatraemia	0		

	Field	Country	Society/ Organisation	National Name	URL	Date Last Search	Search Strategy	Titles	CPG	CPG not devoted to hypo-natraemia
**97**	Nephrology	Cuba	Cuban Society of Nephrologia	Sociedad Cubana de Nefrologia	na	21/12/2013				
**98**	Nephrology	Cyprus	Cypriot Society of Nephrology		na	21/12/2013				
**99**	Nephrology	Czech Republic	Czech Society of Nephrology	Česká nefrologická společnost	http://www.nefrol.cz	21/12/2013	hyponatrémie/hyponatremia/hyponatraemia	0		
**100**	Nephrology	Danmark	Danish Society of Nephrology	Dansk Nefrologisk Selskab	http://www.nephrology.dk	21/12/2013	hyponatriæmi//hyponatremia/hyponatraemia	4	0	
**101**	Nephrology	Egypt	The Egyptian Society of Nephrology & Transplantation	The Egyptian Society of Nephrology & Transplantation	http://www.esnonline.net	21/12/2013	hyponatremia/hyponatraemia	0		
**102**	Nephrology	Equador	Society of Equador of Nephrology	Sociedad Ecuatoriana de Nefrologia	http://sociedadecuatorianadenefrologia.org/	21/12/2013	hiponatremia/hyponatremia/hyponatraemia	0		
**103**	Nephrology	Estonia	Estonian Society of Nephrology		na	21/12/2013				
**104**	Nephrology	Finland	Finnish Society of Nephrology	Suomen Nefrologiyhdistys - Finlands Nefrologförening r.y.	http://www.sny.fi	21/12/2013	hyponatremia/hyponatraemia	0		
**105**	Nephrology	France	French Speaking Society of Dialysis	Société francophone de dialyse	http://www.sfdial.org	21/12/2013	hyponatrémie/hyponatremia/hyponatraemia	0		
**106**	Nephrology	France	French Society of Nephrology	Société Francophone de Néphrologie	http://www.soc-nephrologie.org	21/12/2013	hyponatrémie/hyponatremia/hyponatraemia	35	0	
**107**	Nephrology	Georgia	Dialysis, Nephrology and Kidney Transplantation Union of Georgia		http://dntunion.ge	21/12/2013	hyponatremia/hyponatraemia	2	0	
**108**	Nephrology	Germany	German Society of Nephrology	Deutsche Gesellschaft für Nephrologie	http://www.dgfn.eu/	21/12/2013	hyponatriämie	0		
**109**	Nephrology	Greece	Hellenic Society of Nephrology	Ελληνική Νεφρολογική Εταιρεία	http://www.ene.gr	21/12/2013		0		
**110**	Nephrology	Hong Kong	Hong Kong Society of Nephrology			21/12/2013				

	Field	Country	Society/ Organisation	National Name	URL	Date Last Search	Search Strategy	Titles	CPG	CPG not devoted to hypo-natraemia
**111**	Nephrology	Hungary	Hungarian Society of Nephrology	Magyar Nephrologiai Társaság	http://www.nephrologia.hu	21/12/2013	hyponatraemia/hyponatremia	7	0	
**112**	Nephrology	Iceland	Icelandic Renal Association		na	21/12/2013				
**113**	Nephrology	India	Indian Society of Nephrology		http://www.isn-india.com	21/12/2013				
**114**	Nephrology	Indonesia	The Indonesian Society of Nephrology		na	21/12/2013				
**115**	Nephrology	International	Kidney Diseases Improving Global Outcomes		http://www.kdigo.org/	21/12/2013	hyponatremia/hyponatraemia	3	0	
**116**	Nephrology	International	Latin-American Society of Nephrology and Hypertension	Sodiedad Latino-Americana de Nefrologia e Hypertension	http://www.slanh.org/	21/12/2013				
**117**	Nephrology	International	Panamerican Society for Dialysis & Transplantation		na	21/12/2013				
**118**	Nephrology	International	International Society of Nephrology		http://www.theisn.org	21/12/2013	hyponatraemia/hyponatremia	13	0	
**119**	Nephrology	International	Afraican Association of Nephrology		na	21/12/2013				
**120**	Nephrology	INternational	The Asian Pacific Society of Nephrolo gy		www.apsneph.org	21/12/2013				
**121**	Nephrology	Iran	Iranian Society of Nephrology		http://www.isn-iran.org	21/12/2013	hyponatremia/hyponatraemia/صوديوم نقص الدم	0		
**122**	Nephrology	Ireland	Irish Nephrology Society	Irish Nephrology Society	http://www.nephrology.ie	21/12/2013	hyponatremia/hyponatraemia	0		
**123**	Nephrology	Israel	The Israeli Society of Nephrology and Hypertension		http://www.isnh.org.il	21/12/2013	hyponatremia/hyponatraemia	0		
**124**	Nephrology	Italy	Italian Society of Nephrology	Società Italiana di Nefrologia	http://www.sin-italy.org	21/12/2013		0		
**125**	Nephrology	Japan	Japanese Society of Nephrology		http://www.jsn.or.jp	21/12/2013				
**126**	Nephrology	Jordan	Jordanian Society of Nephrology			21/12/2013				
**127**	Nephrology	Kazakhstan	Kazakhstan Nephrology Association		na	21/12/2013				
**128**	Nephrology	Kenya	Kenya Renal Association		http://www.kenyarenal.org/	21/12/2013				

	Field	Country	Society/ Organisation	National Name	URL	Date Last Search	Search Strategy	Titles	CPG	CPG not devoted to hypo-natraemia
**129**	Nephrology	Korea, South	The Korean Society of Nephrology		http://www.ksn.or.kr/english/	21/12/2013	hyponatraemia/hyponatremia	3	0	
**130**	Nephrology	Kosovo	Kosovo Society of Nephrology		na	21/12/2013				
**131**	Nephrology	Kuwait	The Kuwait Nephrology Association	الكويت جمعية أمراض الكلى	http://www.kna.org.kw	21/12/2013				
**132**	Nephrology	Latvia	Latvian Nephrologist Association		na	21/12/2013				
**133**	Nephrology	Lebanon	Lebanese Society of Nephrology and Hypertension (LSNH)	Societe Libanaise de Nephrologie et d'Hypertension	http://www.lsnh-edu.org/	21/12/2013	hyponatremia/hyponatraemia	0		
**134**	Nephrology	Libya	Libyan Nephrology and Transplantation Society		na	21/12/2013				
**135**	Nephrology	Lithuania	Lithuanian Nephrology, Dialysis and Transplantation Association (LNDTA)	Lietuvos Nefrologijos, Dializes ir Transplantacijos Asociacija	http://www.lndta.lt	21/12/2013	hiponatremija/hyponatremia/hyponatraemia	0		
**136**	Nephrology	Macedonia	Macedonian Society of Nephrology, Dialysis, Transplantation and Artificial Organs		na	21/12/2013				
**137**	Nephrology	Malaysia	Malaysian Society of Nephrology		http://www.msn.org.my	21/12/2013	hyponatraemia/hyponatremia	0		
**138**	Nephrology	Mexico	Mexican Society of Nephrology	Sociedad Mexicana de Nefrologia	http://www.cnm.org.mx	21/12/2013				
**139**	Nephrology	Moldavia	Moldavian Society of Nephrology and Urology		na	21/12/2013				
**140**	Nephrology	Mongolia	Mongolian Society of Nephrology and Urology		na	21/12/2013				
**141**	Nephrology	Montenegro	Montenigrin Society of Nephrology		na	21/12/2013				
**142**	Nephrology	Morocco	Maroccan Society of Nephrology	Societe Marocaine de Nephrologie	http://www.nephro-maroc.org/	21/12/2013				
**143**	Nephrology	Nicaragua	Nicaraguan Society of Nephrology	Sociedad Nicaraguense de Nefrologia	na	21/12/2013				
**144**	Nephrology	Nigeria	Nigerian Association of Nephrology		http://www.nanephrology.org/	21/12/2013				

	Field	Country	Society/ Organisation	National Name	URL	Date Last Search	Search Strategy	Titles	CPG	CPG not devoted to hypo-natraemia
**145**	Nephrology	Norway	Norwegian Society of Nephrology	Norsk Nyremedisinsk Forening	http://www.nephro.no	21/12/2013	hyponatremi/hyponatremia/hyponatraemia	0		
**146**	Nephrology	Pakistan	Pakistan Society of Nephrology and Urology		http://www.psn.com.pk	21/12/2013				
**147**	Nephrology	Palestine	Palestine Society of Nephrology		na	21/12/2013				
**148**	Nephrology	Panama	Panama Society of Nephrology	Sociedad Panamena de Nefrologia	na	21/12/2013				
**149**	Nephrology	Paraguay	Paraguay Society of Nephrology	Sociedad Paraguaya de Nefrologia	na	21/12/2013				
**150**	Nephrology	Peru	Peruan Society of Nephrology	Sociedad Peruana de Nefrologia	www.spn.pe	21/12/2013	hyponatraemia/hyponatremia	0		
**151**	Nephrology	Peurto Rico	Puerto Rican Society of Nephrology	Sociedad de Nefrologia e Hipertens. de Puerto Rico	na	21/12/2013				
**152**	Nephrology	Philippines	Philippine Society of Nephrology		www.e-psn.com	21/12/2013				
**153**	Nephrology	Poland	Polish Society of Nephrology	Polskie Towarzystwo Nefrologiczne	http://www.PTNefro.org	21/12/2013	hyponatremia/hyponatraemia	0		
**154**	Nephrology	Portugal	Portuguese Society of Nephrology	Sociedade Portuguesa de Nefrologia	http://www.spnefro.pt/	21/12/2013	hiponatremia/hyponatremia/hyponatraemia	0		
**155**	Nephrology	Romania	Romanian Society of Nephrology	Societatea Română de Nefrologie	http://www.srnefro.ro/	21/12/2013	hiponatremie/hyponatremia/hyponatraemia	0		
**156**	Nephrology	Russia	Scientific Society of Russian Nephrologists		na	21/12/2013				
**157**	Nephrology	Russia	Russian Dialysis Society	Российское диализное общество	http://www.nephro.ru	21/12/2013	гипонатремия/hyponatremia/hyponatraemia	0		
**158**	Nephrology	Saudi Arabia	Saudi Society of Nephrology and Transplantation		http://www.ssn-sa.com/	21/12/2013				
**159**	Nephrology	Serbia	Serbian Society of Nephrology		na	21/12/2013				
**160**	Nephrology	Singapore	Singapore Society of Nephrology		http://www.ssn.org.sg/	21/12/2013				
**161**	Nephrology	Slovakia	Slovak Nephrological Society	Slovenskej Nefrologickej Spoločnosti	http://www.nefro.sk	21/12/2013	hiponatriemija/hyponatremia/hyponatraemia	0		

	Field	Country	Society/ Organisation	National Name	URL	Date Last Search	Search Strategy	Titles	CPG	CPG not devoted to hypo-natraemia
**162**	Nephrology	Slovenia	Slovenian Society of Nephrology	Slovenskega Nefrološkega Društva	http://www.nephro-slovenia.si/	21/12/2013	hiponatriemija/hyponatremia/hyponatraemia	0		
**163**	Nephrology	South Africa	South African Renal Society		http://www.sa-renalsociety.org/	21/12/2013				
**164**	Nephrology	Spain	Spanish Dialysis and Transplant Society (SEDYT)	Sociedad Española de Diálisis y Trasplante	http://www.sedyt.org	21/12/2013				
**165**	Nephrology	Spain	Spanish Renal Foundation	Fundacion espanola de dialysis	http://fedialisis.com/	21/12/2013				
**166**	Nephrology	Spain	Spanish Society of Nephrology	Sociedad Española de Nefrología	http://www.senefro.org	21/12/2013	hiponatremia/hyponatremia/hyponatraemia	2	0	
**167**	Nephrology	Sweden	Swedish Society of Nephrology	Svensk Njurmedicinsk Förening	http://www.njur.se	21/12/2013	hyponatremi/hyponatremia/hyponatraemia	0		
**168**	Nephrology	Switzerland	Swiss Society of Nephrology	Schweizerische Gesellschaft für Nephrologie	http://www.nephro.ch	21/12/2013	hyponatremia/hyponatraemia	3	0	
**169**	Nephrology	Taiwan	Taiwan Society of Nephrology		http://www.tsn.org.tw/englishVersion	21/12/2013				
**170**	Nephrology	Tanzania	Nephrology Society of Tanzania		http://www.nesot.org/	21/12/2013				
**171**	Nephrology	Thailand	The Nephrology Society of Thailand			21/12/2013				
**172**	Nephrology	The Netherlands	Nederlandse Federatie voor Nefrologie	Dutch Federation of Nephrology	http://www.nefro.nl	21/12/2013	hyponatriëmie/hyponatremia/hyponatraemia	0		
**173**	Nephrology	Tunisia	Tunisian Nephrological Society	Société Tunisienne de Néphrologie	http://www.stn-nephro.org/	21/12/2013				
**174**	Nephrology	Turkey	Turkish Society of Nephrology	Türk Nefroloji Derneği	http://www.tsn.org.tr	21/12/2013	hyponatremi/hyponatremia/hyponatraemia	0		
**175**	Nephrology	Ukraine	Ukranian Nephrology Association		http://www.nephrology.kiev.ua	21/12/2013	гіпонатремія/hyponatremia/hyponatraemia	1	0	
**176**	Nephrology	United Arab Emirates	Emirates Medical Association Nephrology			21/12/2013				

	Field	Country	Society/ Organisation	National Name	URL	Date Last Search	Search Strategy	Titles	CPG	CPG not devoted to hypo-natraemia
**177**	Nephrology	United Kingdom	The Renal Association	The Renal Association	http://www.renal.org	21/12/2013	hyponatremia/hyponatraemia	2	0	
**178**	Nephrology	United Kingdom	Scottish Renal Association			21/12/2013				
**179**	Nephrology	Uruguay	Uruguay Society of Nephrology	Sociedad Uruguaya de Nefrologia	www.nefrouruguay.com	21/12/2013				
**180**	Nephrology	USA	American Society of Nephrology	American Society of Nephrology	http://www.asn-online.org/	21/12/2013				
**181**	Nephrology	Venezuela	Venezuelan Society of Nephrology	Sociedad Venezolana de Nefrologia	http://www.svnefrologia.org/	21/12/2013				
**182**	Endocrinolo gy	Albania	Albanian Medical Society of Endocrinology a nd Diabetology		na	22/12/2013				
**183**	Endocrinolo gy	Algeria	Algerian Society of Endocrinology and Metabolism		na	22/12/2013				
**184**	Endocrinolo gy	Australia/New Zealand	The Endocrine Society of Australia		http://www.endocrinesociety.org.au	22/12/2013	hyponatraemia/hyponatremia	0		
**185**	Endocrinolo gy	Austria	Austrian Society for Endocrinology and Metabolism	Österreichische Gesellschaft für Endokrinologie und Stoffwechsel	http://www.oeges.at/	22/12/2013				
**186**	Endocrinolo gy	Belarus	Belarusian Association of Endocrinologists		na	22/12/2013				
**187**	Endocrinolo gy	Belgium	Belgian Endocrine Society	Belgian Endocrine Society	http://www.endocrinesociety.be/	22/12/2013	hyponatriëmie/hyponatremia/hyponatraemia			
**188**	Endocrinolo gy	Brazil	Brazilian Society of Endocrinology and Metabolism		na	22/12/2013				
**189**	Endocrinolo gy	Bulgaria	Bulgarian Society of Endocrinology		na	22/12/2013				
**190**	Endocrinolo gy	Canada	Canadian Society of Endocrinology and Metabolism		http://www.endo-metab.ca/	22/12/2013				
**191**	Endocrinolo gy	Croatia	Croatian Society for Endocrinology		na	22/12/2013				
**192**	Endocrinolo gy	Cuba	Cuban Society of Endocrinology		na	22/12/2013				

	Field	Country	Society/ Organisation	National Name	URL	Date Last Search	Search Strategy	Titles	CPG	CPG not devoted to hypo-natraemia
**193**	Endocrinology	Cyprus	Cyprus Endocrine Society		na	22/12/2013				
**194**	Endocrinology	Czech Republic	Czech Endocrine Society Czech Republic	České endokrinologické společnosti ČLS JEP	http://www.endokrinologie.cz/	22/12/2013	hyponatrémie/hyponatremia/hyponatraemia	1	0	
**195**	Endocrinology	Danmark	Danish Endocrine Society	Dansk Endokrinologisk Selskab	http://www.endocrinology.dk/	22/12/2013	hyponatriæmi//hyponatremia/hyponatraemia	3	0	
**196**	Endocrinology	Egypt	Egyptian Association of Endocrinology, Diabetes and Atherosclerosi	Egyptian Association of Endocrinology, Diabetes and Atherosclerosi	http://eaeda.org/	22/12/2013	hyponatremia/hyponatraemia	0		
**197**	Endocrinology	Estonia	Estonian Endocrine Society	Eesti Endokrinoloogide Teaduslik Selts	http://www.ees.ee/	22/12/2013				
**198**	Endocrinology	Finland	Finnish Endocrine Society	Terveysportti	http://www.terveysportti.fi/	22/12/2013	hyponatremia/hyponatraemia	50	0	
**199**	Endocrinology	France	French Endocrine Society France	Société Française d'Endocrinologie	http://www.sfendocrino.org/	22/12/2013	hyponatriémie/hyponatremia/hyponatraemia	0		
**200**	Endocrinology	Georgia	Georgian Endocrinologists Society Georgia			22/12/2013				
**201**	Endocrinology	Germany	German Society for Endocrinology Germany	Deutsche Gesellschaft für Endokrinologie	http://www.endokrinologie.net/	22/12/2013	Hyponaträmie/hyponatremia/hyponatraemia	3	0	
**202**	Endocrinology	Greece	Hellenic Endocrine Society Greece	ΕΛΛΗΝΙΚΗ ΕΝΔΟΚΡΙΝΟΛΟΓΙΚΗ ΕΤΑΙΡΕΙΑ	http://www.endo.gr/	22/12/2013	υπονατριαιμία/hyponatremia/hyponatraemia	0		
**203**	Endocrinology	Hong Kong	Hong Kong Society of Endocrinology, Metabolism and Reproduction		http://www.endocrine-hk.org	22/12/2013				
**204**	Endocrinology	Hungary	Hungarian Society of Endocrinology and Metabolism Hungary	Magyar Endokrinológiai és Anyagcsere Társaság	http://www.endokrinologia.hu/	22/12/2013	hyponatraemia/hyponatremia	0		
**205**	Endocrinology	Iceland	Icelandic Endocrine Society	Félag um innkirtlafræði	http://www.innkirtlar.org/	22/12/2013	blóðnatríumlækkun/hyponatraemia/hyponatremia	0		
**206**	Endocrinology	India	Endocrine Society of India		www.endocrinesocietyindia.org	22/12/2013	hyponatraemia/hyponatremia	0		

	Field	Country	Society/ Organisation	National Name	URL	Date Last Search	Search Strategy	Titles	CPG	CPG not devoted to hypo-natraemia
207	Endocrinology	Indonesia	Indonesian Society of Endocrinology	Perkumpulan Endokrinologi Indonesia	http://www.perkeni.org/	22/12/2013				
208	Endocrinology	International	International Pan Arab Critical Care Medicine Society		http://www.ipa-ccms.org/	22/12/2013				
209	Endocrinology	Ireland	Irish Endocrine Society	Irish Endocrine Society	http://www.irishendocrinesociety.ie/	22/12/2013	hyponatremia/hyponatraemia	0		
210	Endocrinology	Israel	Israel Endocrine Society	Israel Endocrine Society	http://www.ies.org.il/	22/12/2013	hyponatremia/hyponatraemia	5	0	
211	Endocrinology	Italy	Italian Endocrine Society	Societa Italiana di Endocrinologia	http://www.societaitalianadiendocrinologia.it/	22/12/2013	ipnatriemia/hyponatremia/hyponatraemia	0		
212	Endocrinology	Italy	Associazione Medici Endocrinologi	Associazione Medici Endocrinology	http://www.associazionemediciendocrinologi.it/	22/12/2013	ipnatriemia/hyponatremia/hyponatraemia	0		
213	Endocrinology	Japan	The Japan Endocrine Society		http://square.umin.ac.jp/endocrine/english/	22/12/2013				
214	Endocrinology	Jordan	Jordanian Society of Endocrinology, Diabetes and Metabolic Diseases		http://medicsorg.tripod.com/jsedm.htm	22/12/2013				
215	Endocrinology	Kuwait	The Kuwaity Society of Endocrinology		http://www.endoq8.com/	22/12/2013				
216	Endocrinology	Latvia	Latvian Association of Endocrinology	Latvijas Endokrinologu Asociācija	http://www.endokrinologi.lv/	22/12/2013	Hiponatriēmija/hyponatremia/hyponatraemia	0		
217	Endocrinology	Lebanon	Libanes Society of Endocrinology, Diabetes and Lipids		http://www.edlsociety.org/	22/12/2013				
218	Endocrinology	Libya	The Libyan Association for Diabetes and Endocrinology Libya	The Libyan Association for Diabetes and Endocrinology Libya	http://www.endodiablibya.org/	22/12/2013	hyponatremia/hyponatraemia/نقص صوديوم الدم	2	0	
219	Endocrinology	Lithuania	Lithuanian Society for Endocrinology			22/12/2013				

	Field	Country	Society/ Organisation	National Name	URL	Date Last Search	Search Strategy	Titles	CPG	CPG not devoted to hypo-natraemia
220	Endocrinology	Macedonia	Macedonian Endocrine Association		http://www.endocrinology.org.mk/	22/12/2013				
221	Endocrinology	Malaysia	Malaysian Endocrine and Metabolic Society		http://www.mems.my/	22/12/2013	hyponatraemia/hyponatremia	0		
222	Endocrinology	Mexico	Mexican Society of Nutrition and Endocrinology	Sociedad Mexicana de Nutrición e Endocrinología	http://www.endocrinologia.org.mx/v2/english/index.php	22/12/2013				
223	Endocrinology	Montenegro	Montenegro, The Endocrinology Association of Montenegro			22/12/2013				
224	Endocrinology	Norway	Norwegian Society of Endocrinology		http://www.endocrinology.no/	22/12/2013				
225	Endocrinology	Pakistan	Pakistan Endocrine Society		http://www.pakendosociety.org/	22/12/2013				
226	Endocrinology	Philippines	Philipine Society of Endocrinology and Metabolism		http://endo-society.org.ph/	22/12/2013				
227	Endocrinology	Poland	Polish Society of Endocrinology	Polskie Towarzystwo Endokrynologiczne	http://www.ptendo.org.pl/	22/12/2013	hiponatremia/hyponatremia/hyponatraemia	3	0	
228	Endocrinology	Portugal	Portuguese Society of Endocrinolgy, Diabetes and Metabolism	Sociedada Portuguese de Endocrinologia Diabetes e Metabolismo	http://www.spedm.org/	22/12/2013	hiponatremia/hyponatremia/hyponatraemia	7	0	
229	Endocrinology	Romania	Romanian Psychoneuroendocrine Society		http://www.rpnes.ro/	22/12/2013				
230	Endocrinology	Romania	Romanian Society of Endocrinology	Societatea Română de Endocrinologie	http://www.sre.ro/	22/12/2013				
231	Endocrinology	Russia	Russian Association of Endocrinologists			22/12/2013				
232	Endocrinology	Saudi Arabia	Suaudi Society of Endocrinlogy and Metabolism		http://www.endocrine.org.sa	22/12/2013	hyponatraemia/hyponatremia	0		
233	Endocrinology	Serbia	Serbian Endocrine Society			22/12/2013				

	Field	Country	Society/ Organisation	National Name	URL	Date Last Search	Search Strategy	Titles	CPG	CPG not devoted to hypo-natraemia
**234**	Endocrinology	Singapore	Endocrine and Metabolic Society of Singapore		http://www.emss.com.sg/	22/12/2013				
**235**	Endocrinology	Slovakia	Slovak Endocrine Society	Slovenská endokrinologická spoločnosť	http://www.endo.sk/	22/12/2013				
**236**	Endocrinology	Slovenia	Slovenian Endocrine Society			22/12/2013	hyponatraemia/hyponatremia	59	0	
**237**	Endocrinology	South Africa	Society for Endocrinology, Metabolism and Diabetes of South Africa		http://www.semdsa.org.za/	22/12/2013				
**238**	Endocrinology	Spain	Spanish Society of Endocrinology and Nutrition		http://www.seen.es/	22/12/2013	hiponatremia/hyponatremia/hyponatraemia	23		
**239**	Endocrinology	Sweden	Swedish Endocrine Society			22/12/2013				
**240**	Endocrinology	Switzerland	Swiss Society of Endocrinology and Diabetes	Schweizerische Gesellschaft für Endokrinologie und Diabetologie	http://www.sgedssed.ch/	22/12/2013	Hyponatriämie/hyponatremia/hyponatraemia	0		
**241**	Endocrinology	The Netherlands	Netherlands Society for Endocrinology	Nederlandse Vereniging voor Endocrinologie	http://www.nve.nl	22/12/2013	hyponatiëmie/hyponatremia/hyponatraemia	2	0	
**242**	Endocrinology	Tunisia	Tunisian Society of Endocrinology			22/12/2013				
**243**	Endocrinology	Turkey	Turkey Society of Endocrinology and Metabolism			22/12/2013				
**244**	Endocrinology	Ukraine	Association of Endocrinologists of the Ukraine		http://iem.kiev.ua/	22/12/2013	гіпонатремія/hyponatremia/hyponatraemia	0		
**245**	Endocrinology	United Kingdom	Society for Endocrinology	Society for Endocrinology	http://www.endocrinology.org/	22/12/2013				
**246**	Endocrinology	Uruguay	Uruguayan Society of Endocrinology and Metabolism		http://www.endosuem.org.uy/	22/12/2013				
**247**	Endocrinology	USA	The Endocrine Society		https://www.endocrine.org/	22/12/2013	hyponatraemia/hyponatremia	19	0	
**248**	Endocrinology	USA	American Association of Clinical Endocrinologists		https://www.aace.com/	22/12/2013	hyponatraemia/hyponatremia	4	0	

	Field	Country	Society/ Organisation	National Name	URL	Date Last Search	Search Strategy	Titles	CPG	CPG not devoted to hypo-natraemia
249	Endocrinology	Venezuela	Venezuelan Society of Endocrinology and Metabolism		na	22/12/2013				
250	Intensive Care Medicine	Albania	Albanian Society of Anaesthesia and Intensive Care		na	22/12/2013				
251	Intensive Care Medicine	Argentina	Argentinian Society of Intensive Care	Sociedad Argentina de Terapia Intensiva	http://www.sati.org.ar/	22/12/2013	hyponatremia/hyponatraemia	0		
252	Intensive Care Medicine	Armenia	Armenian Society of Anaesthesiologists and Intensive Care Specialists		http://freenet.am/~armanest/index.htm	22/12/2013				
253	Intensive Care Medicine	Australia/New Zealand	Australian & New Zealand Intensive Care Society	Australian & New Zealand Intensive Care Society	http://www.anzics.com.au/	22/12/2013	hyponatremia/hyponatraemia	0		
254	Intensive Care Medicine	Austria	Austrian Society for Anesthesiology, Reanimation and Intensive Care Medicine	Österreichische Gesellschaft fur Anasthesiologie, Reanimation und Intensivmedizin	http://www.oegari.at/	22/12/2013		0		
255	Intensive Care Medicine	Bangladesh	Bangladesh Society of Critical Care Medicine	Bangladesh Society of Critical Care Medicine	http://www.bsccm.net/	22/12/2013	hyponatremia/hyponatraemia	0		
256	Intensive Care Medicine	Belarus	BALARUS - Belarusian Society of Anaethetists and Experts in Resuscitation		http://www.baics.org/	22/12/2013				
257	Intensive Care Medicine	Belgium	Belgian Society of Intensive Care & Emergency Medicine	Belgische Vereniging voor Intensieve Geneeskunde/Société belge de Médecine Intensive	http://www.siz.be/	22/12/2013	hyponatriëmie/hyponatremia/hyponatraemia	0		
258	Intensive Care Medicine	Belize	Belize Critical Care Society		na	22/12/2013		0		
259	Intensive Care Medicine	Bolivia	Bolivian Society fo Critical Medicine and Intensive Therapy	Sociedad Boliviana de Medicina Critica Y Terapia Intensiva	na	22/12/2013		0		
260	Intensive Care Medicine	Bosnia and Herzegovina	Society of Intensive Care Medicine of Bosnia-Herzigovina		na	22/12/2013				

	Field	Country	Society/ Organisation	National Name	URL	Date Last Search	Search Strategy	Titles	CPG	CPG not devoted to hypo-natraemia
**261**	Intensive Care Medicine	Brazil	Brazilian Association of Intensive Care Medicine	Asociacao de Medicina Intensiva Brasileira	http://www.amib.org.br/	22/12/2013	hyponatremia/hiponatremia	0		
**262**	Intensive Care Medicine	Canada	Canadian Critical Care Society	Canadian Critical Care Society	http://www.canadiancriticalcare.org/	22/12/2013	hyponatremia/hyponatraemia	0		
**263**	Intensive Care Medicine	Chile	Chilean Society of Intensive Care Medicine	Sociedad Chilena de Medicina Intensiva	http://www.medicina-intensiva.cl/web/	22/12/2013		0		
**264**	Intensive Care Medicine	China	Chinese Society of Critical Care Medicine		na	22/12/2013		0		
**265**	Intensive Care Medicine	China	Society of Critical Care Medicine of the Chinese Association of Integration of Traditional Medicine & Modern Medicine		na	22/12/2013		0		
**266**	Intensive Care Medicine	Colombia	Colombian Association of Critical Medicine and Intensive Care	Asociacion Colombiana de Medicina Critica y Cuidado Intensivo	http://www.amci.org.co	22/12/2013	hyponatrémie/hyponatremia/hyponatraemia	0		
**267**	Intensive Care Medicine	Costa Rica	Costa Rican Society of Intensive Care Medicine	Asociacion de Medicina Intensiva de Costa Rica	na	22/12/2013		0		
**268**	Intensive Care Medicine	Croatia	Croatian Society for Emergency and Medical Intensive Care Medicine	Hrvatsko društvo za hitnu i internističku intenzivnu medicinu	http://www.hdhiim.org/	22/12/2013				
**269**	Intensive Care Medicine	Czech Republic	Czech Society of Anesthesiology & Intensive Care Medicine	Ceska spolecnost anestesiologie resisitace a intenzivni mediciny	http://www.csarim.cz/	22/12/2013	hyponatrémie/hyponatremia/hyponatraemia	5	0	
**270**	Intensive Care Medicine	Czech Republic	Czech Society of Intensive Care Medicine	Česka společnost intenzivní medicíny	http://www.csim.cz/	22/12/2013	hyponatrémie/hyponatremia/hyponatraemia	5	0	
**271**	Intensive Care Medicine	Danmark	Danish Society of Intensive Care Therapy		http://www.dsit.dk/	22/12/2013				
**272**	Intensive Care Medicine	Denmark	Danish Society of Anaesthesiology and Intensive Care Medicine	Dansk Selskab for Anæstesiologi og Intensiv Medicin	http://www.dasaim.dk	22/12/2013				

	Field	Country	Society/ Organisation	National Name	URL	Date Last Search	Search Strategy	Titles	CPG	CPG not devoted to hypo-natraemia
**273**	Intensive Care Medicine	Egypt	Egyptian Society of Critical Care and Emergency		na	22/12/2013		0		
**274**	Intensive Care Medicine	Equador	Equatorian Society of Anaesthesiology and Critical Medicine	Sociedad Ecuatoriana de Medicina Critica	na	22/12/2013		0		
**275**	Intensive Care Medicine	Estonia	Estonian Society of Anaesthesiologists		http://www.anest.ee/	22/12/2013				
**276**	Intensive Care Medicine	Finland	Finnish Society of Intensive Care	Finnish Society of Intensive Care	http://www.fimnet.fi/	22/12/2013	hyponatremia/hyponatraemia	0		
**277**	Intensive Care Medicine	France	French Speaking Society of Reanimation	Societe de Reanimation de Langue Francaise	http://www.srlf.org/	22/12/2013	hyponatrémie/hyponatremia/hyponatraemia	113	0	
**278**	Intensive Care Medicine	Georgia	Georgian Society of Anesthesiology and Critical Care Medicine		http://www.gsaccm.ge/	22/12/2013	hyponatremia/hyponatraemia	0		
**279**	Intensive Care Medicine	Georgia	Association of Catastrophe & Critical Care Medicine of Georgia			22/12/2013		0		
**280**	Intensive Care Medicine	Germany	German Interdisciplinary Society of Intensive Care Medicine	Deutsche Interdisziplinare Vereinigung fur Intensivmedizin	http://www.divi-org.de/	22/12/2013	Hyponatriämie/hyponatremia/hyponatraemia	0		
**281**	Intensive Care Medicine	Greece	Greek Society of Intensive Care	ΕΛΛΗΝΙΚΗ ΕΤΑΙΡΕΙΑ ΕΝΤΑΤΙΚΗΣ ΘΕΡΑΠΕΙΑΣ	http://www.icu.gr/	22/12/2013	υπονατριαιμία/hyponatremia/hyponatraemia	0		
**282**	Intensive Care Medicine	Hong Kong	Hong Kong Society of Critical Care Medicine		http://www.hksccm.org/	22/12/2013		0		
**283**	Intensive Care Medicine	Hungary	Hungarian Society of Anaesthesiology and Intensive Therapy	Magyar Aneszteziológiai és Intenzív Terápiás Társaság	http://www.aneszinfo.hu	22/12/2013	hyponatraemia/hyponatremia	1	0	
**284**	Intensive Care Medicine	India	Indian Society of Critical Care Medicine	Indian Society of Critical Care Medicine	http://www.isccm.org/	22/12/2013	hyponatremia/hyponatraemia	6	0	
**285**	Intensive Care Medicine	India	National Association of Critical Care Medicine		na	22/12/2013		0		

	Field	Country	Society/ Organisation	National Name	URL	Date Last Search	Search Strategy	Titles	CPG	CPG not devoted to hypo-natraemia
**286**	Intensive Care Medicine	Indonesia	Indonesian Society of Critical Care Medicine	Perhimpunan Dokter Intensive Care Indonesia	http://www.perdici.org/	22/12/2013	hiponatremia/hyponatremia/hyponatraemia	0		
**287**	Intensive Care Medicine	Ireland	Intensive Care Society of Ireland	Intensive Care Society of Ireland	http://www.icmed.com/	22/12/2013	hyponatremia/hyponatraemia	0		
**288**	Intensive Care Medicine	Israel	Israel Society of Critical Care Medicine		na	22/12/2013		0		
**289**	Intensive Care Medicine	Italy	Italian Society of Intensice Care	Societa Italiana di Terapia Intensiva	na	22/12/2013		0		
**290**	Intensive Care Medicine	Italy	Italian Society of Aneastthesia Analgesia Reanimation and Intensive Care	Società Italiana di Anestesia Analgesia Rianimazione e Therapia Intensiva	http://www.siaarti.it	22/12/2013	hiponatremia/hyponatremia/hyponatraemia	0		
**291**	Intensive Care Medicine	Japan	Japanese Society of Intensive Care Medicine		http://www.jsicm.org	22/12/2013	低ナトリウム血症/hyponatremia/hyponatraemia	29	0	
**292**	Intensive Care Medicine	Korea, South	Korean Society of Critical Care Medicine		http://www.ksccm.org	22/12/2013	hyponatremia/hyponatraemia	0		
**293**	Intensive Care Medicine	Korea, South	Korean Society of Critical Care Medicine		na	22/12/2013				
**294**	Intensive Care Medicine	Kosovo	Association of Kosovar Anaesthesiologists		na	22/12/2013				
**295**	Intensive Care Medicine	Kuwait	Kuwait Society of Intensive Care			22/12/2013				
**296**	Intensive Care Medicine	Latvia	Latvian Society of Intensive Care & Emergency Medicine		na	22/12/2013		0		
**297**	Intensive Care Medicine	Lebanon	Lebanese Society of Critical Care		na	22/12/2013		0		
**298**	Intensive Care Medicine	Libya	Libyan Society of Anaesthesia, Intensive Care and Resuscitation		na	22/12/2013				
**299**	Intensive Care Medicine	Lithuania	Lithuanian Society of Anaesthesiology & Intensive Care	Lietuvos Anesteziologų – Reanimatologų draugija	http://anest-rean.lt/	22/12/2013				

	Field	Country	Society/ Organisation	National Name	URL	Date Last Search	Search Strategy	Titles	CPG	CPG not devoted to hypo-natraemia
300	Intensive Care Medicine	Macedonia	Macedonian Society of Anesthesia & Intensive Care	Macedonian Society of Anesthesia & Intensive Care	http://www.msa.org.mk/	22/12/2013	hyponatremia/hyponatraemia	0		
301	Intensive Care Medicine	Malaysia	Malaysian Society of Intensive Care		na	22/12/2013		0		
302	Intensive Care Medicine	Mexico	Mexican College of Critical Medicine	Colegio Mexicano de Medicina Critica	http://www.commec.org.mx/	22/12/2013		0		
303	Intensive Care Medicine	Mongolia	Mongolian Society of Anaesthesiology and Intensive Care		na	22/12/2013		0		
304	Intensive Care Medicine	Morocco	Maroccan Society of Anaesthesiology and Intensice Care	Soieté Marocaine d'Anesthésie et de Réanimation	http://www.smar.ma	22/12/2013				
305	Intensive Care Medicine	Norway	Norway Society of Anesthiology	Norsk Anestesiologisk Forening	http://legeforeningen.no	22/12/2013		0		
306	Intensive Care Medicine	Pakistan	Pakistan Society of Intensive Care		na	22/12/2013		0		
307	Intensive Care Medicine	Panama	Panama Association Critical Medicine and Care	Asociacion Panamena de Medicina Critica y Terapia	na	22/12/2013		0		
308	Intensive Care Medicine	Peru	Peruvian Society of Intensive Care	Sociedad Peruana de Medicina Intensiva	na	22/12/2013		0		
309	Intensive Care Medicine	Philippines	Philippine Society of Anesthesiologists		http://www.psa-ph.org/	22/12/2013	hyponatremia/hyponatraemia	0		
310	Intensive Care Medicine	Philippines	Philippine Society of Critical Care Medicine		na	22/12/2013		0		
311	Intensive Care Medicine	Poland	Polish Society of Anaesthesiology & Intensive Care		http://www.ptaiit.org/	22/12/2013				
312	Intensive Care Medicine	Portugal	Portugese Society of Intensive Care	Sociedade Portuguesa de Cuidados Intensivos	na	22/12/2013		0		
313	Intensive Care Medicine	Romania	Romanean Society of Anesthesiology and Intensive Care	Societatea Romana de Anestezie-Terapie Intensiva	http://www.srati.ro/	22/12/2013	hyponatremia/hyponatraemia	0		

	Field	Country	Society/ Organisation	National Name	URL	Date Last Search	Search Strategy	Titles	CPG	CPG not devoted to hypo-natraemia
**314**	Intensive Care Medicine	Russia	Society of Anaesthesiologists and reanimatologists of Central Russia		na	22/12/2013		0		
**315**	Intensive Care Medicine	Saudi Arabia	Saudi Critical Care Society		na	22/12/2013		0		
**316**	Intensive Care Medicine	Saudi Arabia	Seudi Society of Internal Medicine		http://ssim.kau.edu.sa	22/12/2013				
**317**	Intensive Care Medicine	Serbia	Serbian Society of Intensive Care Medicine			22/12/2013				
**318**	Intensive Care Medicine	Singapore	Society of Intensive Care Medicine		na	22/12/2013		0		
**319**	Intensive Care Medicine	Slovakia	Slovak Society of Anesthesia & Intensive Care Medicine		http://www.ssaim.sk/	22/12/2013		0		
**320**	Intensive Care Medicine	Slovenia	Slovenian Society of Intensive Care Medicine	Slovensko Združenje za Intensivno Medico	http://www.szim.si/	22/12/2013	hiponatriemija/hyponatremia/hyponatraemia	0		
**321**	Intensive Care Medicine	South Africa	Critical Care Society of Southern Africa		na	22/12/2013		0		
**322**	Intensive Care Medicine	Spain	Spanish Society Intensive Care Medicine	Sociedad Espanola de Medicina Intensiva, Critica y Unidades Coronarias	http://www.semicyuc.org/	22/12/2013	hiponatremia/hyponatremia/hyponatraemia	0		
**323**	Intensive Care Medicine	Sudan	Sudan Society of Anesthesiologists		na	22/12/2013		0		
**324**	Intensive Care Medicine	Sweden	Swedish Society of Anesthesiology and Intensive Care	Svensk Forening for Anestesi och Intensivvard	http://www.sfai.se/	22/12/2013	hyponatremi/hyponatremia/hyponatraemia	3	0	
**325**	Intensive Care Medicine	Switzerland	Swiss Society of Intensive Care Medicine	Societe Suisse de Medecine Intensive	http://www.swiss-icu.ch/	22/12/2013	Hyponaträmie/hyponatremia/hyponatraemia	0		
**326**	Intensive Care Medicine	Taiwan	Society of Emergency and Critical Care Medicine		http://www.seccm.org.tw/English.asp	22/12/2013				
**327**	Intensive Care Medicine	The Netherlands	Dutch Society of Intensive Care	Nederlands Vereniging vor Intensive Care	http://nvic.nl	22/12/2013	hyponatriëmie/hyponatremia/hyponatraemia	0		

	Field	Country	Society/ Organisation	National Name	URL	Date Last Search	Search Strategy	Titles	CPG	CPG not devoted to hypo-natraemia
**328**	Intensive Care Medicine	Tunisia	Societé Tunisienne d'Anesthésie et de Réanimation		http://www.staartunisie.org/home.php	22/12/2013	hyponatriémie/hyponatremia/hyponatraemia	0		
**329**	Intensive Care Medicine	Turkey	Turkish Society of Medical and Surgical Intensive Care Medicine	Türk Dahili ve Cerrahi Bilimler	http://www.dcyogunbakim.org.tr/	22/12/2013		0		
**330**	Intensive Care Medicine	Ukraine	Ukranian Society of Anaesthesiologists	АСОЦІАЦІЯ АНЕСТЕЗІОЛОГІВ УКРАЇНИ	http://www.aay.org.ua	22/12/2013				
**331**	Intensive Care Medicine	United Kingdom	The Intensive Care Society	The Intensive Care Society	http://www.ics.ac.uk/	22/12/2013	hyponatremia/hyponatraemia	3	0	
**332**	Intensive Care Medicine	United Kingdom	Scottish Intensive Care Society		http://www.scottishintensivecare.org.uk/	22/12/2013				
**333**	Intensive Care Medicine	Uruguay	Uruguay Society of Intensive Care Medicine	Sociedad Uruguaya de Medicina Intensiva	http://www.sumi.org.uy	22/12/2013		0		
**334**	Intensive Care Medicine	USA	Society of Critical Care Medicine	Society of Critical Care Medicine	http://www.sccm.org	22/12/2013	hyponatremia	2	0	
**335**	Intensive Care Medicine	Venezuela	Venezuelan Society of Critical Medicine	Sociedad Venezolana de Medicina Critica	http://www.svmc.com.ve/	22/12/2013		0		
**336**	Intensive Care Medicine		Slovenian Society of Anaesthesiology and Intensive Care	Slovensko združenje za anesteziologijo in intenzivno medicino	http://www.szaim.org	22/12/2013				
**337**	Intensive Care Medicine		Jordan Society of Anaesthesia and Intensive Care		http://www.jsaic.org/	22/12/2013				
**338**	not applicable	not applicable	-	-	www.google.com	23/12/2013	(Hyponatraemia OR hyponatremia) AND Guideline	100	1	
