# Supplementary material for: Diagnosis and treatment of hyponatremia: a systematic review of clinical practice guidelines and consensus statements
Source: BMC Med. 2014 Dec 11;12:1. doi: 10.1186/s12916-014-0231-1 (PMC4276109; doi:10.1186/s12916-014-0231-1)
Supplement: Additional file 2: Table S2. — Data extraction template. [file 12916_2014_231_MOESM2_ESM.pdf]

Additional file 2: Table S2. Data Extraction Template

Item	Entry
Guideline Id	
Guideline Organisation/society= Developer	
Name of guideline or consensus statement	
Year of publication	
Funding source	
Country	
Target population	
Definition of hyponatraemia	
Target Users	
Guideline writers	
Guideline Review	
Guideline Update	
Methods Support	
Evidence Base	
Level of Evidence	
Grade recommendations	
Recommendation 1	
Recommendation 2	
Recommendation 3	
Recommendation 4	
Recommendation 5	
Recommendation 6	
Recommendation 7	
Recommendation 8	
Recommendation 9	
Recommendation 10	
