# Supplementary material for: Diagnosis and treatment of hyponatremia: a systematic review of clinical practice guidelines and consensus statements
Source: BMC Med. 2014 Dec 11;12:1. doi: 10.1186/s12916-014-0231-1 (PMC4276109; doi:10.1186/s12916-014-0231-1)
Supplement: Additional file 3: Table S3. — Structure and content of the AGREE instrument. [file 12916_2014_231_MOESM3_ESM.pdf]

Additional file 3: Table S3. Structure and Content of the AGREE instrument

The following is adapted from the AGREE instrument (AGREE Collaboration. Appraisal of guidelines research and evaluation. London UK: The AGREE Collaboration at www. Agreecollaboration.org, 2001.)

Domain	Content	No. of items
Scope and purpose	Addresses the overall aim of the guideline, the specific clinical questions and the target patient population	3
Stakeholder involvement	Addresses the extent to which the guideline represents the views of its intended users (relevant professional groups, patients, target users defined, piloting among target users)	4
Rigour of development	Addresses the process used to collect and synthesize the evidence, the methods to formulate the recommendations, process for updating the guidelines, external review	7
Clarity and presentation	Addresses the language and format of the guideline (recommendations are specific and unambiguous, different options for management are presented, key recommendations are identifiable, tools for application are available)	4
Applicability	Addresses the likely organisational, behavioural, and cost implications of applying the guideline, key criteria for monitoring and/or audit purposes	3
Editorial independence	Addresses the independence of the recommendations and acknowledgement of possible conflict of interest from the guideline development group	2
