# Supplementary material for: Diagnosis and treatment of hyponatremia: a systematic review of clinical practice guidelines and consensus statements
Source: BMC Med. 2014 Dec 11;12:1. doi: 10.1186/s12916-014-0231-1 (PMC4276109; doi:10.1186/s12916-014-0231-1)
Supplement: Additional file 4: Table S4. — Table of excluded studies. [file 12916_2014_231_MOESM4_ESM.pdf]

Additional file 4: Table S4. Table of Excluded Studies

Author	Year	Reason for exclusion	Reference
**Oster**	1994	Related to the treatment of hyponatraemia but not an evidence-based clinical practice guideline or a consensus statement on the treatment of hyponatraemia – this includes a conference or discussion paper or personal opinion.	[1]
**Oh**	1995	Related to the treatment of hyponatraemia but not an evidence-based clinical practice guideline or a consensus statement on the treatment of hyponatraemia, – this includes a conference or discussion paper or personal opinion.	[2]
**Soupart**	1996	Related to the treatment of hyponatraemia but not an evidence-based clinical practice guideline or a consensus statement on the treatment of hyponatraemia – this includes a conference or discussion paper or personal opinion.	[3]
**Gowrishankar**	1998	Related to the treatment of hyponatraemia but not an evidence-based clinical practice guideline or a consensus statement on the treatment of hyponatraemia – this includes a conference or discussion paper or personal opinion.	[4]
**American College of Sports Medicine**	2000	Not related to the treatment of hyponatraemia.	[5]
**Han**	2002	Related to the treatment of hyponatraemia but not an evidence-based clinical practice guideline or a consensus statement on the treatment of hyponatraemia – this includes a conference or discussion paper or personal opinion.	[6]
**Janicic**	2003	Related to the treatment of hyponatraemia but not an evidence-based clinical practice guideline or a consensus statement on the treatment of hyponatraemia – this includes a conference or discussion paper or personal opinion.	[7]
**Brouh**	2004	Related to the treatment of hyponatraemia but not an evidence-based clinical practice guideline or a consensus statement on the treatment of hyponatraemia – this includes a conference or discussion paper or personal opinion.	[8]
**Culpepper**	2004	Related to the treatment of hyponatraemia but not an evidence-based clinical practice guideline or a consensus statement on the treatment of hyponatraemia – this includes a conference or discussion paper or personal opinion.	[9]
**Hsieh**	2004	Related to the treatment of hyponatraemia but not an evidence-based clinical practice guideline or a consensus statement on the treatment of hyponatraemia - this includes a conference or discussion paper or personal opinion.	[10]
**Thomas**	2004	Related to the treatment of hyponatraemia but not an evidence-based clinical practice guideline or a consensus statement on the treatment of hyponatraemia – this includes a conference or discussion paper or personal opinion.	[11]
**Hew-Butler**	2005	Related to the treatment of hyponatraemia and an evidence-based clinical practice guideline or a consensus statement on the treatment of hyponatraemia, published in English, French, German, Dutch, Spanish or Italian. However this is an older version of an updated guideline, which was replaced by updated recommendations.	[12]
**Kraft**	2005	Related to the treatment of hyponatraemia but not an evidence-based clinical practice guideline or a consensus statement on the	[13]

		treatment of hyponatraemia – this includes a conference or discussion paper or personal opinion.	
**Hew-Butler**	2006	Not related to the treatment of hyponatraemia.	[14]
**Roberts**	2007	Not related to the treatment of hyponatraemia.	[15]
**Verbalis**	2007	Related to the treatment of hyponatraemia and an evidence-based clinical practice guideline or a consensus statement on the treatment of hyponatraemia, published in English, French, German, Dutch, Spanish or Italian. However this is an older version of an updated guideline, which was replaced by updated recommendations.	[16]
**Beltrami**	2008	Not related to the treatment of hyponatraemia.	[17]
**Esposito**	2011	Related to the treatment of hyponatraemia but not an evidence-based clinical practice guideline or a consensus statement on the treatment of hyponatraemia – this includes a conference or discussion paper or personal opinion.	[18]
**Gomez Navarro**	2011	Related to the treatment of hyponatraemia but not an evidence-based clinical practice guideline or a consensus statement on the treatment of hyponatraemia – this includes a conference or discussion paper or personal opinion.	[19]
**Human**	2011	Related to the treatment of hyponatraemia but not an evidence-based clinical practice guideline or a consensus statement on the treatment of hyponatraemia – this includes a conference or discussion paper or personal opinion.	[20]
**Josiassen**	2011	Related to the treatment of hyponatraemia but not an evidence-based clinical practice guideline or a consensus statement on the treatment of hyponatraemia – this includes a conference or discussion paper or personal opinion.	[21]
**Hoorn**	2013	Related to the treatment of hyponatraemia and evidence-based clinical practice guideline but duplicate of already included document.	[22]
**Tzamaloukas**	2013	Related to the treatment of hyponatraemia but not an evidence-based clinical practice guideline or a consensus statement on the treatment of hyponatraemia – this includes a conference or discussion paper or personal opinion.	[23]
**Bennett**	2013	Related to the treatment of hyponatraemia and a clinical practice guideline, but to very specific case of exercise associated hyponatraemia in very remote areas where measurement of serum sodium concentrations mostly not available.	[24]
**Lander**	2013	Not related to the treatment of hyponatraemia, but rather to its prevention.	[25]
**Laville**	2013	Related to the treatment of hyponatraemia but not an evidence-based clinical practice guideline or a consensus statement on the treatment of hyponatraemia – but rather multiple cases to highlight the difficulty in diagnosis and treatment of hyponatraemia.	[26]
**Combs**	2014	Related to the treatment of hyponatraemia but not an evidence-based clinical practice guideline or a consensus statement on the treatment of hyponatraemia – this includes a conference or discussion paper or personal opinion.	[27]
**Lamas**	2014	Related to the treatment of the syndrome of inappropriate antidiuretic hormone secretion but not an evidence-base clinical practice guideline or consensus statement, but rather a review article.	[28]

**Lee**	2014	Related to the treatment of the syndrome of inappropriate antidiuretic hormone secretion but not an evidence-base clinical practice guideline or consensus statement, but rather a review article.	[29]

References

1. Oster JR, Singer I: **Hyponatremia: Focus on therapy**. *South Med J* 1994, **87**(12):1195-1202.
2. Oh MS, Kim HJ, Carroll HJ: **Recommendations for treatment of symptomatic hyponatremia**. *Nephron* 1995, **70**(2):143-150.
3. Soupart A, Decaux G: **Therapeutic recommendations for management of severe hyponatremia: current concepts on pathogenesis and prevention of neurologic complications**. *Clin Nephrol* 1996, **46**(3):149-169.
4. Gowrishankar M, Lin SH, Mallie JP, Oh MS, Halperin ML: **Acute hyponatremia in the perioperative period: insights into its pathophysiology and recommendations for management**. *Clin Nephrol* 1998, **50**(6):352-360.
5. American College of Sports Medicine, American Dietetic Association, Dietitians of Canada: **Joint Position Statement: nutrition and athletic performance**. *Med Sci Sport Exerc* 2000, **32**(12):2130-2145.
6. Han D-S, Cho B-S: **Therapeutic approach to hyponatremia**. *Nephron* 2002, **92 Suppl 1**:9-13.
7. Janicic N, Verbalis JG: **Evaluation and management of hypo-osmolality in hospitalized patients**. *Endocrinol Metabol Clin North Am* 2003, **32**(2):459-481.
8. Brouh Y, Paut O, Tsimaratos M, Camboulives J: **[Postoperative hyponatremia in children: Pathophysiology, diagnosis and treatment]**. *Ann Fr Anesth* 2004, **23**(1):39-49.
9. Culpepper RM, Clements BD, Pence SR: **Hypertonic saline: Patterns of and guidelines for use**. *South Med J* 1994, **87**(12):1203-1207.
10. Hsieh M: **Recommendations for treatment of hyponatraemia at endurance events**. *Sport Med* 2004, **34**(4):231-238.
11. Thomas L, Kwok Y, Edelman MJ: **Management of paraneoplastic syndromes in lung cancer**. *Curr Treat Option On* 2004, **5**(1):51-62.
12. Hew-Butler T, Almond C, Ayus JC, Dugas J, Meeuwisse W, Noakes T, Reid S, Siegel A, Speedy D, Stuempfle K *et al*: **Consensus statement of the 1st International Exercise-Associated Hyponatremia Consensus Development Conference, Cape Town, South Africa 2005**. *Clin J Sport Med* 2005, **15**(4):208-213.
13. Kraft MD, Btaiche IF, Sacks GS, Kudsk KA: **Treatment of electrolyte disorders in adult patients in the intensive care unit**. *Am J Health-Syst Ph* 2005, **62**(16):1663-1682.
14. Hew-Butler T, Verbalis JG, Noakes TD: **Updated fluid recommendation: Position statement from the International Marathon Medical Directors Association (IMMDA)**. *Clin J Sport Med* 2006, **16**(4):283-292.
15. Roberts WO: **Fractured fairy tales: hyponatraemia and the American College of Sports Medicine fluid recommendations**. *Brit J Sport Med* 2007, **41**(2):109; author reply 109-111.
16. Verbalis JG, Goldsmith SR, Greenberg A, Schrier RW, Sterns RH: **Hyponatremia treatment guidelines 2007: expert panel recommendations**. *Am J Med* 2007, **120**(11 Suppl 1):S1-21.
17. Beltrami FG, Hew-Butler T, Noakes TD: **Drinking policies and exercise-associated hyponatraemia: Is anyone still promoting overdrinking?** *Brit J Sport Med* 2008, **42**(10):496-501.
18. Esposito P, Piotti G, Bianzina S, Malul Y, Dal Canton A: **The syndrome of inappropriate antidiuresis: Pathophysiology, clinical management and new therapeutic options**. *Nephron - Clin Pract* 2011, **119**(1):c62-c73.

19. Gomez Navarro L, De Arriba De La Fuente G: **Diagnostic and treatment protocol of hyponatremia. [Spanish] Protocolo diagnostico y tratamiento de la hiponatremia**. *Medicine* 2011, **10**(80):5438-5440.
20. Human T: **Current therapeutic options for hyponatremia: Indications, limitations, and confounding variables**. *Pharmacotherapy* 2011, **31**(5 Part 2):18S-24S.
21. Josiassen RC: **Hyponatremia in psychosis: Treatment guidelines and future directions**. *Biol Psychiat* 2011, **1)**:208S.
22. Hoorn EJ, Tuut MK, Hoorntje SJ, van Saase JLMC, Zietse R, Geers AB: **Dutch guideline for the management of electrolyte disorders - 2012 revision**. *Neth J Med* 2013, **71**(3):153-165.
23. Tzamaloukas AH, Malhotra D, Rosen BH, Raj DSC, Murata GH, Shapiro JI: **Principles of management of severe hyponatremia**. *J Am Heart Ass* 2013, **2**(1):e000240.
24. Bennett BL, Hew-Butler T, Hoffman MD, Rogers IR, Rosner MH, Wilderness Medical S: **Wilderness Medical Society practice guidelines for treatment of exercise-associated hyponatremia**. *Wild Environ Med* 2013, **24**(3):228-240.
25. Lander A: **Paediatric fluid and electrolyte therapy guidelines**. *Surgery* 2013, **31**(12):599-602.
26. Laville M, Burst V, Peri A, Verbalis JG: **Hyponatremia secondary to the syndrome of inappropriate secretion of antidiuretic hormone (SIADH): Therapeutic decision-making in real-life cases**. *Clin Kidney J* 2013, **6**(Suppl 1):i1-i20.
27. Combs S, Berl T: **Dysnatremias in patients with kidney disease**. *Am J Kidney Dis* 2014, **63**(2):294-303.
28. Lamas C, Del Pozo C, Villabona C: **Clinical guidelines for management of diabetes insipidus and syndrome of inappropriate antidiuretic hormone secretion after pituitary surgery**. *Endocrinol Nutric* 2014, **61**(4):e15-e24.
29. Lee JJY, Kilonzo K, Nistico A, Yeates K: **Management of hyponatremia**. *Can Med Assoc J* 2014, **186**(8):e281-e286.
