# Supplementary material for: Diagnosis and treatment of hyponatremia: a systematic review of clinical practice guidelines and consensus statements
Source: BMC Med. 2014 Dec 11;12:1. doi: 10.1186/s12916-014-0231-1 (PMC4276109; doi:10.1186/s12916-014-0231-1)
Supplement: Additional file 5: Table S5. — Mean scores across reviewers for the individual AGREE II domain items. [file 12916_2014_231_MOESM5_ESM.pdf]

Additional file 5: Table S5. Mean scores across reviewers for the individual AGREE II domain items

	Domain 1. Scope and Purpose			Domain 2. Stakeholder involvement			Domain 3. Rigour of Development								Domain 4. Clarity and Presentation			Domain 5. Applicability				Domain 6. Editorial independence	
	I1	I2	I3	I4	I5	I6	I7	I8	I9	I10	I11	I12	I13	I14	I15	I16	I17	I18	I19	I20	I21	I22	I23
Guideline																							
NIV	6.3	5.3	5.0	4.0	1.5	5.3	6.0	6.3	4.8	1.5	5.0	5.0	2.8	2.8	4.5	5.8	5.0	5.0	6.3	4.0	5.0	6.0	5.5
NHS	6.8	3.8	5.8	1.0	1.3	6.8	4.8	1.5	1.5	1.5	4.3	4.5	1.0	6.8	5.5	4.5	6.3	1.3	2.3	1.3	2.5	3.0	1.3
GAIN*	1.8	1.5	5.0	5.3	1.0	1.0	1.0	1.0	1.0	1.0	2.0	1.0	1.0	4.8	3.3	3.3	5.5	1.0	3.5	1.0	2.5	3.3	1.0
AEEH*	2.8	1.8	5.5	1.8	1.0	2.5	1.3	1.0	1.3	1.3	1.5	1.5	1.5	1.0	3.5	2.0	5.5	1.5	1.5	1.3	2.3	1.3	1.0
EHN*	3.5	3.3	5.3	5.3	1.0	2.3	1.3	1.0	1.0	1.3	2.8	1.8	2.0	1.0	3.5	2.5	3.3	2.5	4.3	1.0	2.5	1.0	2.5
ERBP/ESE/ESICM	6.5	6.5	6.5	5.0	3.5	6.3	6.3	6.5	5.5	5.8	5.8	5.8	5.8	6.5	6.0	5.8	4.8	5.3	5.3	3.5	3.5	5.8	4.5
UF	6.5	6.5	6.3	6.0	1.3	2.5	4.0	2.8	3.3	2.5	2.8	3.8	1.3	1.0	2.8	4.8	5.5	1.3	3.3	1.0	2.0	3.3	5.3
HEP	3.0	3.5	1.5	3.5	1.0	1.0	3.0	1.0	1.8	1.3	2.8	2.0	3.0	1.0	2.0	3.0	3.0	1.5	1.8	1.0	2.5	1.0	2.8
RCHM*	2.5	1.8	4.5	1.5	1.3	1.5	1.5	1.3	1.0	2.0	2.0	1.3	1.5	6.3	5.3	1.8	3.8	1.5	2.0	1.0	4.5	3.0	1.0
EAH-ICD*	6.8	6.8	5.3	6.8	3.5	5.0	2.8	1.3	1.5	2.0	4.8	4.5	1.3	1.5	4.3	4.0	1.8	2.5	6.0	1.0	3.8	5.0	1.0

NIV – Nederlandse Internisten Vereniging [16]; NHS – National Health Service [17]; GAIN - Guidelines and Audit Implementations Network [22] ; AEEH – La Asociación Española para el Estudio del Hígado [23]; EHN – European Hyponatraemia Network [25]; ERBP – European Renal Best Practice, ESE – European Society of Endocrinology, ESICM – European Society of Intensive Care Medicine [20]; UF – University of Florida [18]; HEP – Hyponatremia Expert Panel [19]; RCH Melbourne – the Royal Children’s Hospital Melbourne [21]; EAH-ICD – International Exercise-Associated Hyponatremia Consensus Development Conference [24]; [Na]: serum sodium concentration;* classified as consensus statement
